# Use of hormone replacement therapy and risk of venous thromboembolism: nested case-control studies using the QResearch and CPRD databases

**DOI:** 10.1136/bmj.k4810

**Published:** 2019-01-09

**Authors:** Yana Vinogradova, Carol Coupland, Julia Hippisley-Cox

**Affiliations:** 1 Division of Primary Care, University of Nottingham, Nottingham NG2 7RD, UK

## Abstract

**Objective:**

To assess the association between risk of venous thromboembolism and use of different types of hormone replacement therapy.

**Design:**

Two nested case-control studies.

**Setting:**

UK general practices contributing to the QResearch or Clinical Practice Research Datalink (CPRD) databases, and linked to hospital, mortality, and social deprivation data.

**Participants:**

80 396 women aged 40-79 with a primary diagnosis of venous thromboembolism between 1998 and 2017, matched by age, general practice, and index date to 391 494 female controls.

**Main outcome measures:**

Venous thromboembolism recorded on general practice, mortality, or hospital records. Odds ratios were adjusted for demographics, smoking status, alcohol consumption, comorbidities, recent medical events, and other prescribed drugs.

**Results:**

Overall, 5795 (7.2%) women who had venous thromboembolism and 21 670 (5.5%) controls had been exposed to hormone replacement therapy within 90 days before the index date. Of these two groups, 4915 (85%)****and 16 938 (78%) women used oral therapy, respectively, which was associated with a significantly increased risk of venous thromboembolism compared with no exposure (adjusted odds ratio 1.58, 95% confidence interval 1.52 to 1.64), for both oestrogen only preparations (1.40, 1.32 to 1.48) and combined preparations (1.73, 1.65 to 1.81). Estradiol****had a lower risk than conjugated equine oestrogen for oestrogen only preparations (0.85, 0.76 to 0.95) and combined preparations (0.83, 0.76 to 0.91). Compared with no exposure, conjugated equine oestrogen with medroxyprogesterone acetate had the highest risk (2.10, 1.92 to 2.31), and estradiol with dydrogesterone had the lowest risk (1.18, 0.98 to 1.42). Transdermal preparations were not associated with risk of venous thromboembolism, which was consistent for different regimens (overall adjusted odds ratio 0.93, 95% confidence interval 0.87 to 1.01).

**Conclusions:**

In the present study, transdermal treatment was the safest type of hormone replacement therapy when risk of venous thromboembolism was assessed. Transdermal treatment appears to be underused, with the overwhelming preference still for oral preparations.

## Introduction

Venous thromboembolism (VTE) is a rare but serious risk associated with hormone replacement therapy (HRT). HRT is used to prevent a range of symptoms experienced by many women during the menopause, such as hot flushes and night sweats. In 2015, in response to a halving of HRT use after two large studies^1^ ^2^ had raised concerns about the safety profile of HRT (including VTE risk), the National Institute for Health and Care Excellence (NICE) published its first guideline on diagnosis and management of menopausal symptoms in the United Kingdom.^3^ A central theme was the need to inform women of the risks and benefits of HRT so that they can make appropriate treatment choices; but the recommendations relate to overall use of HRT, distinguishing only between oral and transdermal preparations.^3^ The guideline recommends further research on the risks of HRT containing different types of progestogens in combination with oestrogen. The guideline also notes that the VTE risk appears greater for oral preparations than for transdermal treatment. The guideline is likely to result in an increase in HRT use in women with menopausal symptoms, increasing the need for detailed studies of the long term risks of different HRT regimens.

Oral HRT formulations can be oestrogen only (unopposed) using conjugated equine oestrogen or estradiol, or oestrogen combined with a progestogen (opposed). Progestogens in combined formulations include medroxyprogesterone acetate or newer agents such as norgestrel, dydrogesterone, or drospirenone. Previous studies assessing the VTE risk associated with different HRT treatments either have not distinguished between the types of oestrogen or progestogen, or were powered to analyse only the most common preparations.^4^ ^5^ Findings from randomised controlled trials summarised in a Cochrane systematic review^4^ were based mainly on the Women’s Health Initiative trial of women in the United States, who were predominantly in relatively good health.^6^ The review reported that increased risk was associated with oestrogen only oral preparations, and with oral combinations of conjugated equine oestrogen and medroxyprogesterone acetate. Other preparations have become available in the past 20 years and across other countries, but observational studies on these treatments, which have been summarised in a meta-analysis, did not have consistent definitions of outcome and were not sufficiently powered to investigate individual types of HRT.^5^ Therefore, there is insufficient information on VTE risk associated with specific HRT formulations for clinicians and women to make informed choices about treating menopausal symptoms.

Our case-control study, based on the general female population in the UK, aimed to assess the associations between VTE risk and all available types of HRT in the UK between 1998 and 2017. The study performed additional analyses of subgroups of women based on age and body mass index.

## Methods

### Study design

Full details of the study design have been published elsewhere.^7^ In summary, we conducted nested case-control studies using the two UK primary care research databases QResearch and Clinical Practice Research Datalink (CPRD) and included all practices, which had contributed data for at least a year. We identified two open cohorts of women aged 40-79 years and registered with study practices between January 1998 and February 2017. We excluded women with previous records of VTE or with less than one year of medical records.

### Selection of cases and controls

For the QResearch database, we identified cases of incident VTE recorded between January 1998 and February 2017 in the general practice records, or in hospital admissions or mortality records. For the CPRD analysis, we identified cases by using only the general practice records. We used incidence density sampling for both databases to match each case to up to five controls from the same practice and by year of birth.^8^ The first date of diagnosis of VTE for cases became the index date for matched controls.

### Exposure to hormone replacement therapy

We used HRT prescription information for the last year before the index date and included types of oestrogen and progestogen, dosage, and duration of exposure. We defined overall exposure to HRT as any exposure to oral or transdermal (patch, subcutaneous, or gel formulation) preparations containing estradiol. Oral and transdermal exposures were analysed separately. We identified a few treatments that included both tablets and patches; in such instances, the patient was considered to have been exposed to both oral and transdermal HRT. Exposure was categorised as recent (within 90 days before the index date), past (91-365 days), or no exposure. The study’s main focus was on recent exposure to HRT, because a previous study showed that past exposure is not associated with increased risk of VTE.^9^ All included women had records for the one year before the index date; therefore, if women had no HRT prescriptions in this period, it meant that they were not exposed in terms of this study definition.

We classified exposure based on the most recent HRT prescription in the 90 days before the index date. Oral HRT included oestrogen only preparations (conjugated equine oestrogen and estradiol) and combined preparations (oestrogen with medroxyprogesterone acetate, dydrogesterone, norethisterone acetate, norgestrel/levonorgestrel, or drospirenone). Because of low numbers of participants exposed to norgestrel/levonorgestrel or to drospirenone, we analysed these preparations as one type of drug—that is, other progestogens structurally related to testosterone.^10^ Transdermal HRT included oestrogen only and combined estradiol and we analysed this route of administration separately. We removed participants from the analysis if they had prescriptions for two different types of oestrogen or progestogen in the last 90 days issued on the same date. A few women had switched to another HRT within the last 90 days, so we added a switch indicator to the analysis.

We also analysed different regimens—cyclical or continuous—overall and separately for preparations with a sufficient number of cases (estradiol combined with norethisterone, and estradiol combined with dydrogesterone).

The dose was categorised as low (≤0.625 mg for oral conjugated equine oestrogen, ≤1 mg for oral estradiol, ≤50 μg for transdermal estradiol) or high. We assessed exposure duration in the year before the index date by adding up the days of prescriptions plus any periods between prescriptions of shorter than 90 days. Duration was categorised as short term (≤84 days) or long term (>84 days). No use of HRT in the past year was the reference category for all analyses.

Our analysis also included other preparations with oestrogen or progestogen that are used as topical (cream) or vaginal (pessaries) treatments. In addition, we included two other drugs that do not contain oestrogen: tibolone for menopausal symptoms and raloxifene for osteoporosis.

### Confounders

We adjusted the analyses for confounding factors, which might have influenced whether doctors prescribed HRT or what specific HRT treatment was chosen. These factors are listed in table 1 and include lifestyle factors such as smoking status and body mass index, family history of VTE, comorbidities, and acute conditions associated with increased VTE risk.^11^ Comorbidities had to be recorded at any time before the index date, and included asthma, atrial fibrillation, cancer, cardiovascular disease, chronic obstructive pulmonary disease, chronic renal disease, coagulation disturbances, congestive cardiac failure, inflammatory bowel disease, rheumatoid arthritis, systemic lupus erythematosus, and varicose veins. We considered acute conditions to be confounders if they were recorded in the six months before the index date; these conditions were gall bladder surgery, hip fracture or hip replacement operation, pregnancy, respiratory infection, and urinary tract infection. The analysis also included hospital admissions between two and six months before the index date. Other drugs that could be prescribed to women using HRT were included, either as current use (within 90 days before the index date) or past use (91-365 days before the index date). These were antipsychotics, antidepressants (tricyclic, selective serotonin reuptake inhibitors, and others), aspirin, tamoxifen, oral contraceptives, and progestogen only preparations.

**Table 1:** Characteristics of study population at or before the index date by database (QResearch and CPRD). Values are percentages (numbers) unless stated otherwise

	QResearch			CPRD	
	Cases (n=52 137)	Controls (n=259 542)		Cases (n=28 259)	Controls (n=131 952)
Age:					
Mean (SD)	63.8 (11.0)	63.8 (11.0)		63.8 (10.9)	64.0 (10.8)
40-54	23.3 (12 128)	23.3 (60 484)		22.9 (6467)	22.3 (29 463)
55-64	22.5 (11 721)	22.5 (58 392)		23.2 (6566)	23.2 (30 652)
65-79	54.3 (28 288)	54.2 (140 666)		53.9 (15 226)	54.4 (71 837)
Mean (SD) years of records	9.3 (5.7)	9.4 (5.7)		9.4 (5.7)	10.5 (5.8)
Ethnicity:					
Recorded	68.6 (35 766)	74.0 (192 107)		70.1 (19 799)	63.9 (84 293)
White or not recorded	94.8 (49 435)	94.2 (244 422)		97.7 (27 600)	97.7 (128 854)
Bangladeshi	0.2 (86)	0.3 (899)		0.0 (10)	0.0 (57)
Black African	0.8 (409)	0.8 (2141)		0.3 (93)	0.3 (349)
Caribbean	1.5 (777)	1.2 (3044)		0.5 (148)	0.4 (479)
Chinese	0.1 (44)	0.2 (596)		0.0 (13)	0.1 (191)
Indian	1.0 (518)	1.2 (2998)		0.4 (120)	0.5 (707)
Other	0.9 (491)	1.0 (2667)		0.6 (170)	0.6 (750)
Other Asian	0.3 (176)	0.6 (1473)		0.2 (47)	0.2 (323)
Pakistani	0.4 (201)	0.5 (1302)		0.2 (58)	0.2 (242)
Townsend deprivation fifth*:					
Most affluent	18.7 (9756)	21.6 (56 152)		14.3 (4053)	15.7 (20 738)
2	19.5 (10 187)	21.2 (55 110)		14.2 (4024)	14.9 (19 654)
3	21.2 (11 043)	20.8 (54 101)		12.9 (3649)	12.6 (16 655)
4	21.1 (11 020)	19.4 (50 290)		11.9 (3355)	10.7 (14 177)
Most deprived	19.4 (10 131)	16.9 (43 889)		7.8 (2207)	6.7 (8905)
Body mass index:					
Recorded	86.4 (45 069)	85.7 (222 326)		90.0 (25 433)	87.5 (115 395)
Mean (SD)	29.3 (6.4)	27.3 (5.5)		29.5 (6.8)	27.4 (5.6)
15-24	23.8 (12 410)	33.4 (86 623)		24.9 (7040)	33.7 (44 414)
25-29	27.3 (14 239)	29.3 (76 056)		29.7 (8405)	30.9 (40 770)
≥30	35.3 (18 420)	23.0 (59 647)		35.3 (9988)	22.9 (30 211)
Smoking status:					
Recorded	94.6 (49 316)	93.6 (242 821)		96.6 (27 291)	95.0 (125 306)
None	41.4 (21 582)	44.6 (115 657)		55.9 (15 789)	59.2 (78 169)
Former	36.9 (19 224)	34.1 (88 558)		24.2 (6838)	20.6 (27 193)
Light (1-9 cigarettes/day)	8.8 (4588)	8.1 (21 067)		7.1 (2019)	6.4 (8447)
Moderate (10-19)	4.4 (2299)	4.3 (11 156)		5.3 (1509)	5.4 (7060)
Heavy (≥20)	3.1 (1623)	2.5 (6383)		4.0 (1136)	3.4 (4437)
Alcohol use:					
Recorded	85.4 (44 521)	84.6 (219 463)		87.6 (24 758)	86.4 (114 067)
None	26.2 (13 645)	23.0 (59 656)		35.5 (10 034)	31.9 (42 044)
Former use	12.6 (6571)	10.6 (27 564)		2.5 (706)	1.8 (2346)
Trivial (<1 unit/day)	30.3 (15 809)	32.0 (83 003)		31.7 (8961)	32.4 (42 766)
Light (1-2)	9.1 (4752)	11.1 (28 746)		12.6 (3560)	14.9 (19 696)
Moderate (3-6)	6.5 (3378)	7.5 (19 346)		3.9 (1109)	4.3 (5632)
Heavy (7-9)	0.4 (185)	0.3 (702)		0.9 (264)	0.8 (1099)
Very heavy (≥10)	0.3 (181)	0.2 (446)		0.4 (124)	0.4 (484)
Chronic conditions:	55.6 (28 967)	35.3 (91 526)		57.9 (16 349)	36.7 (48 366)
Asthma	15.4 (8041)	11.2 (28 980)		16.6 (4702)	12.0 (15 838)
Atrial fibrillation	3.2 (1691)	2.5 (6569)		3.8 (1078)	2.6 (3461)
Cancer	20.9 (10 873)	6.7 (17 419)		21.0 (5936)	6.9 (9069)
Cardiovascular disease	12.6 (6558)	8.8 (22 791)		14.4 (4076)	10.0 (13 174)
Chronic obstructive pulmonary disease	7.0 (3641)	3.8 (9925)		6.8 (1932)	3.8 (4956)
Chronic renal disease	8.5 (4407)	5.4 (14 137)		8.5 (2401)	5.5 (7229)
Congestive cardiac failure	2.9 (1507)	1.4 (3547)		3.3 (923)	1.6 (2048)
Inflammatory bowel disease	1.7 (890)	1.0 (2506)		1.8 (501)	1.0 (1277)
Rheumatoid arthritis	3.6 (1890)	2.0 (5171)		4.1 (1159)	2.0 (2679)
Systemic lupus erythematosus	0.4 (220)	0.2 (429)		0.4 (120)	0.2 (238)
Varicose veins	7.0 (3634)	5.2 (13 430)		7.7 (2173)	5.4 (7111)
Conditions and hospital admission in previous six months:	25.4 (13 250)	11.5 (29 813)		28.6 (8096)	11.4 (15 087)
Gall bladder surgery	0.4 (229)	0.2 (392)		0.4 (109)	0.1 (181)
Hip fracture/operation	3.3 (1743)	0.4 (961)		3.6 (1015)	0.3 (369)
Hospital admission	6.6 (3460)	1.7 (4376)		7.7 (2185)	0.7 (873)
Pregnancy	0.6 (320)	0.3 (854)		0.5 (132)	0.2 (302)
Respiratory infection	12.6 (6571)	6.4 (16 672)		13.6 (3838)	7.2 (9488)
Urinary infection	6.8 (3555)	3.7 (9477)		7.0 (1985)	3.7 (4857)
Other characteristics:					
Early menopause	0.3 (151)	0.3 (792)		0.3 (79)	0.3 (334)
Family history of VTE	0.0 (20)	0.0 (32)		0.2 (57)	0.1 (102)
Oophorectomy/hysterectomy	28.1 (14 647)	23.7 (61 600)		30.7 (8682)	25.1 (33 068)
Other drugs in previous 90 days:					
Antipsychotics	3.4 (1750)	1.2 (3024)		3.3 (943)	1.0 (1377)
Aspirin	13.4 (7010)	11.1 (28 743)		14.0 (3963)	10.8 (14 218)
Combined oral contraceptives	0.8 (433)	0.3 (871)		1.4 (405)	0.9 (1216)
Oral progestogen	0.8 (429)	0.4 (1157)		0.8 (222)	0.4 (539)
Other antidepressants	3.7 (1918)	1.6 (4167)		3.2 (892)	1.5 (1949)
Selective serotonin reuptake inhibitors	10.2 (5341)	6.5 (16 949)		4.2 (1186)	2.5 (3343)
Tamoxifen	3.7 (1917)	1.2 (3077)		2.3 (642)	0.7 (869)
Tricyclic antidepressants	9.8 (5129)	6.1 (15 757)		9.5 (2698)	5.5 (7264)

CPRD=Clinical Practice Research Datalink; SD=standard deviation; VTE=venous thromboembolism.

* Based on linked cases and controls.

### Statistical analysis

We used conditional logistic regression adjusted for the confounders to estimate odds ratios and to assess associations between HRT exposure and VTE risk. We assumed missing values for body mass index, smoking status, and alcohol consumption were missing at random and used imputation by chained equations. We created 10 imputed datasets and the imputation model included all listed confounders, current and past exposure, and the case-control indicator. We combined the odds ratios from each imputed dataset using Rubin’s rule.^12^

We conducted analyses of QResearch and CPRD separately, but tried to keep the study designs as similar as possible (identical when data availability allowed). Adjusted estimates from the databases were combined by a meta-analysis technique. The findings were consistently similar between the databases; we did not expect or detect any heterogeneity. Therefore, we used a fixed effect model to combine the results of the two analyses, and we report only combined adjusted odds ratios in the text and figures. We present adjusted odds ratios for the separate QResearch and CPRD analyses in the tables and supplementary tables.

We used the number needed to harm to estimate the magnitude of VTE risk in women exposed to oral HRT^13^; this was based on the adjusted odds ratios from the combined analysis and the VTE rate in the unexposed population. We obtained this rate using CPRD data by following the cohort until the first prescription of HRT. Because exposure to HRT is highest in women aged 55-64 and VTE risk increases with age, we calculated the overall risk and the risk by age. To account for multiple comparisons, we chose a 1% significance level. We calculated 95% confidence intervals to allow comparison with other studies. Stata version 15 was used for all analyses.

### Additional analyses

We ran three sensitivity analyses to address a number of assumptions. Firstly, to assess the assumption that previous use of anticoagulants was not related to an unrecorded VTE event, we ran an analysis excluding all women with previous exposure to anticoagulants. Secondly, we conducted a sensitivity analysis because of a difference in the data sources between QResearch and CPRD. All QResearch practices are linked to hospital admissions, mortality, and Townsend deprivation data whereas only 56% of CPRD practices (61% of included patients) are linked. For the main analysis, we used data from all the CPRD practices, but for this sensitivity analysis we only included linked CPRD practices. We also excluded participants with previous VTE events on hospital records from this second sensitivity analysis. Thirdly, to assess the plausibility of the missing at random assumption, we performed an analysis on women with complete data for body mass index, smoking status, and alcohol consumption.

For ease of comparisons with other studies, we conducted four additional analyses. Firstly, we ran an analysis on women with a VTE diagnosis supported by hospital admissions or mortality records, or with anticoagulant prescriptions six weeks before or after the VTE diagnosis. We used only practices with linked data for this analysis. Secondly, we ran an additional analysis on idiopathic participants who did not have any of the comorbidities or recent medical events associated with an increased risk of VTE. We also conducted two subgroup analyses using two clinically important variables to stratify risk: age (categories 40-54, 55-64, 65-79 years), and body mass index (categories: not overweight or obese, <25; overweight, 25-30; and obese, >30). These analyses investigated whether associations differed among the subgroups.

### Patient and public involvement

This study was unfunded, so patient and public involvement initially envisaged in anticipation of funding was not possible. No patients were involved in setting the research question or the outcome measures, nor were they involved in developing plans for design or implementation of the study. No patients were asked to aid in interpreting or disseminating the results. There are no plans to disseminate the results of the research to the relevant patient community.

## Results

**Fig 1:**
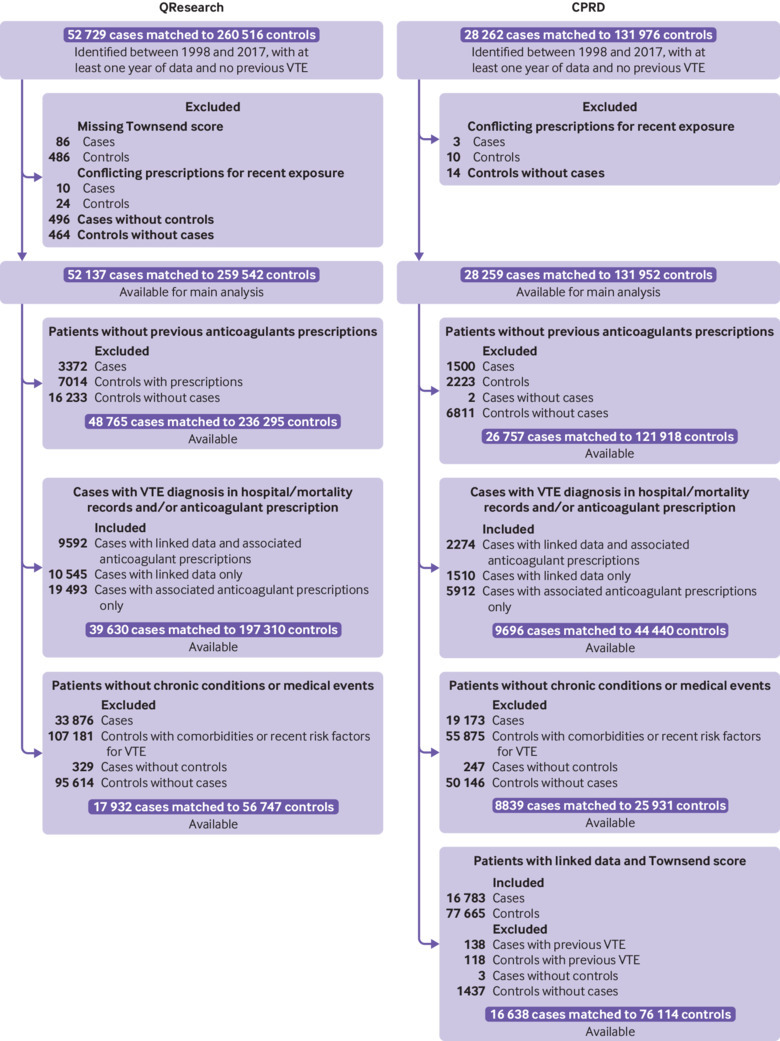
Flow of included cases and controls for QResearch and Clinical Practice Research Datalink (CPRD) analyses with numbers excluded and reasons for exclusion. VTE=venous thromboembolism

We identified 52 137 cases from the QResearch database between 2 January 1998 and 5 February 2017 (the latest available data linkage date) using general practice, hospital admissions, or mortality records. We identified 28 259 cases from the CPRD database between 2 January 1998 and 22 February 2017 using general practice records. Of the CPRD cases, 16 638 were also linked to hospital admissions and mortality data between 2 January 1998 and 31 March 2016; these cases were used in a sensitivity analysis (fig 1).

Table 1 presents the characteristics of the study participants across the databases. More than half of the women who had VTE were aged 65 or older; they were also more likely to have comorbidities than controls (overall, 56% *v* 36%), such as cancer (21% *v* 7%), cardiovascular disease (13% *v* 9%), or chronic renal disease (8% *v* 5%). Women who had VTE were more likely than controls to have recent medical events than controls (27% *v* 12%), such as respiratory or urinary infection (20% *v* 10%), hip fracture or operation (3.4% *v* 0.3%), or hospital admission (7% *v* 1%), and to use antidepressants (24% *v* 14%; table 1).

### Exposure (main analysis)

**Fig 2:**
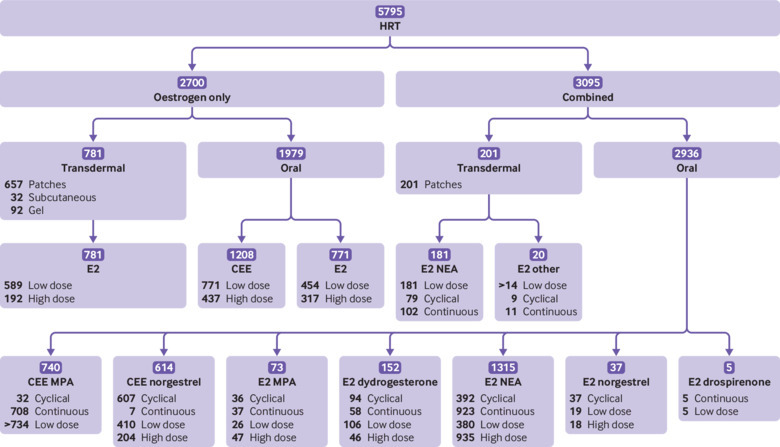
Hormone replacement therapy (HRT) preparations available in the UK and number of women with venous thromboembolism exposed to HRT from QResearch and Clinical Practice Research Datalink (CPRD) databases. Some treatments comprised tablets and patches; 60 women in the oestrogen only group and 42 in the combined group were prescribed these treatments. CEE=conjugated equine oestrogen; E2=estradiol; MPA=medroxyprogesterone acetate; NEA=norethisterone acetate; other=dydrogesterone or levonorgestrel

When combining CPRD and QResearch results, we found that 5795 (7.2%) women with VTE and 21 670 (5.5%) controls were exposed to HRT in the 90 days before the index date. Figure 2 presents all available preparations and the numbers of exposed cases (for controls supplementary eFigure 1). In women exposed to HRT, 4915 (85%) cases and 16 938 (78%) controls used oral preparations, including 102 (1.8%) cases and 312 (1.4%) controls who also had transdermal preparations; 880 (14%) cases and 4731 (19%) controls used transdermal HRT only. Most of the transdermal preparations were prescribed in the form of patches (87% (n=858) in cases, 88% (n=4460) in controls), with only small proportions of women having subcutaneous and gel preparations (fig 2 and supplementary eFigure 1). 

Supplementary eTable 1 presents the number of study participants unexposed and exposed to oral and transdermal HRT across the confounding factors to highlight differences in prescribing. Women in the two younger age groups were more likely to have been exposed to HRT than women in the oldest group. Women exposed to HRT were less likely to be obese and have comorbidities such as cardiovascular disease, chronic renal disease, and cancer, but more likely to have used antidepressants. Women using transdermal HRT were more likely to have had oophorectomy or hysterectomy than women on oral HRT (73% (n=714) cases and 67% (n=3407) controls *v* 45% (n=2185) cases and 43% (n=7278) controls); these women were also slightly older and had more comorbidities (supplementary eTable 1).

Table 2 shows the number of study participants exposed to all types of HRT and adjusted odds ratios by database and for the combined analysis, compared with no exposure (unadjusted odds ratios are presented in supplementary eTable 2). Table 3 presents information for direct comparisons between different types of HRT. Overall exposure to HRT in the past 90 days was associated with a 43% increased VTE risk (adjusted odds ratio 1.43, 95% confidence interval 1.38 to 1.48; table 2) compared with no HRT use in the past year. Use of oral preparations was associated with a significantly increased VTE risk (1.58, 1.52 to 1.64), whereas transdermal HRT was not associated with VTE risk (0.93, 0.87 to 1.01; table 2). Compared with transdermal HRT, oral HRT was associated with a 70% increased risk of VTE (1.70, 1.56 to 1.85; table 3).

**Table 2:** Exposure to different types of HRT and adjusted odds ratios for venous thromboembolism risk by database and combined analysis

	QResearch			CPRD		Combined analysis Odds ratio (95% CI)	P
	No of cases/ controls	Adjusted odds ratio (95% CI)*		No of cases/ controls	Adjusted odds ratio (95% CI)*		
Any HRT	3769/14 604	1.43 (1.37 to 1.50)		2026/7066	1.43 (1.35 to 1.52)	1.43 (1.38 to 1.48)	<0.001
Oral preparations	3207/11 338	1.59 (1.52 to 1.67)		1708/5600	1.56 (1.46 to 1.67)	1.58 (1.52 to 1.64)	<0.001
Oestrogen only:	1297/4568	1.42 (1.32 to 1.52)		682/2213	1.36 (1.23 to 1.50)	1.40 (1.32 to 1.48)	<0.001
CEE	786/2692	1.50 (1.37 to 1.64)		422/1308	1.48 (1.31 to 1.68)	1.49 (1.39 to 1.60)	<0.001
E2	511/1876	1.31 (1.17 to 1.46)		260/905	1.20 (1.02 to 1.40)	1.27 (1.16 to 1.39)	<0.001
Combined with progestogen	1910/6770	1.73 (1.63 to 1.84)		1026/3387	1.72 (1.58 to 1.86)	1.73 (1.65 to 1.81)	<0.001
CEE combined:	883/2870	1.90 (1.75 to 2.07)		471/1407	1.94 (1.72 to 2.18)	1.91 (1.79 to 2.05)	<0.001
CEE MPA	501/1438	2.22 (1.99 to 2.48)		239/732	1.87 (1.59 to 2.20)	2.10 (1.92 to 2.31)	<0.001
CEE norgestrel	382/1432	1.59 (1.41 to 1.80)		232/675	2.00 (1.70 to 2.36)	1.73 (1.57 to 1.91)	<0.001
E2 combined:	1027/3900	1.61 (1.49 to 1.74)		555/1980	1.56 (1.40 to 1.74)	1.59 (1.49 to 1.69)	<0.001
E2 MPA	51/215	1.51 (1.09 to 2.09)		24/106	1.21 (0.74 to 1.96)	1.44 (1.09 to 1.89)	0.01
E2 dydrogesterone	101/520	1.19 (0.95 to 1.50)		51/259	1.15 (0.82 to 1.60)	1.18 (0.98 to 1.42)	0.09
E2 norethisterone	848/3043	1.69 (1.56 to 1.84)		467/1570	1.65 (1.46 to 1.86)	1.68 (1.57 to 1.80)	<0.001
E2 other progestogens†	27/122	1.40 (0.90 to 2.17)		13/45	1.65 (0.85 to 3.22)	1.42 (1.00 to 2.03)	0.05
Regimen:							
Combined cyclical	1123/3606	1.96 (1.82 to 2.11)		411/1362	1.70 (1.50 to 1.92)	1.55 (1.44 to 1.66)	<0.001
Combined continuous	787/3164	1.48 (1.36 to 1.62)		825/2878	1.73 (1.56 to 1.92)	1.88 (1.77 to 1.99)	<0.001
E2 dydrogesterone cyclical	38/208	1.13 (0.78 to 1.63)		31/147	1.15 (0.75 to 1.76)	1.21 (0.95 to 1.53)	0.1
E2 dydrogesterone continuous	63/312	1.23 (0.92 to 1.64)		20/112	1.14 (0.68 to 1.91)	1.13 (0.84 to 1.53)	0.4
E2 norethisterone cyclical	585/1963	1.85 (1.67 to 2.04)		129/461	1.49 (1.20 to 1.85)	1.44 (1.28 to 1.63)	<0.001
E2 norethisterone continuous	263/1080	1.42 (1.23 to 1.65)		338/1109	1.72 (1.49 to 1.98)	1.80 (1.66 to 1.95)	<0.001
Oral oestrogen dose:							
CEE ≤0.625 mg	498/1934	1.37 (1.23 to 1.53)		273/891	1.47 (1.26 to 1.71)	1.40 (1.28 to 1.53)	<0.001
CEE >0.625 mg	288/758	1.81 (1.56 to 2.11)		149/417	1.51 (1.22 to 1.86)	1.71 (1.51 to 1.93)	<0.001
E2 ≤1 mg	303/1201	1.25 (1.09 to 1.43)		151/549	1.17 (0.96 to 1.44)	1.22 (1.09 to 1.37)	<0.001
E2 >1 mg	208/675	1.41 (1.19 to 1.67)		109/356	1.23 (0.97 to 1.57)	1.35 (1.18 to 1.55)	<0.001
CEE ≤0.625 mg norgestrel	262/1094	1.45 (1.26 to 1.68)		148/521	1.68 (1.38 to 2.06)	1.53 (1.36 to 1.72)	<0.001
CEE >0.625 mg norgestrel	120/338	2.06 (1.64 to 2.58)		84/154	3.06 (2.28 to 4.10)	2.38 (1.99 to 2.85)	<0.001
E2 ≤1 mg dydrogesterone	69/384	1.11 (0.85 to 1.46)		37/203	1.14 (0.78 to 1.66)	1.12 (0.90 to 1.40)	0.3
E2 >1 mg dydrogesterone	32/136	1.40 (0.93 to 2.12)		14/56	1.18 (0.61 to 2.28)	1.34 (0.94 to 1.90)	0.1
E2 ≤1 mg norethisterone	236/1082	1.39 (1.20 to 1.62)		144/586	1.37 (1.11 to 1.68)	1.38 (1.23 to 1.56)	<0.001
E2 >1 mg norethisterone	612/1961	1.85 (1.68 to 2.05)		323/984	1.81 (1.57 to 2.08)	1.84 (1.69 to 2.00)	<0.001
Transdermal preparations:	640/3519	0.92 (0.84 to 1.00)		342/1525	0.97 (0.85 to 1.11)	0.93 (0.87 to 1.01)	0.07
E2 only	503/2646	0.94 (0.85 to 1.04)		278/1204	0.99 (0.86 to 1.15)	0.96 (0.88 to 1.04)	0.3
E2 combined	137/873	0.84 (0.69 to 1.01)		64/321	0.91 (0.67 to 1.23)	0.86 (0.73 to 1.01)	0.06
Combined cyclical	71/462	0.84 (0.64 to 1.10)		22/124	0.91 (0.55 to 1.49)	0.85 (0.67 to 1.09)	0.2
Combined continuous	66/411	0.84 (0.63 to 1.10)		42/197	0.89 (0.61 to 1.30)	0.86 (0.69 to 1.06)	0.2
Transdermal oestrogen dose:							
E2 ≤50 μg	377/2110	0.91 (0.81 to 1.02)		212/970	0.99 (0.84 to 1.16)	0.94 (0.85 to 1.03)	0.2
E2 >50 μg	126/536	1.06 (0.86 to 1.31)		66/234	1.01 (0.75 to 1.38)	1.05 (0.88 to 1.24)	0.6
Other menopausal treatment:							
Tibolone	224/1218	0.99 (0.85 to 1.16)		144/641	1.07 (0.87 to 1.31)	1.02 (0.90 to 1.15)	0.8
Raloxifene	114/419	1.48 (1.18 to 1.86)		66/212	1.51 (1.10 to 2.06)	1.49 (1.24 to 1.79)	<0.001
CEE cream	22/126	0.98 (0.61 to 1.57)		31/135	1.09 (0.71 to 1.69)	1.04 (0.76 to 1.43)	0.8
E2 vaginal	87/545	0.84 (0.66 to 1.07)		204/1128	0.85 (0.71 to 1.00)	0.84 (0.73 to 0.97)	0.02

CEE=conjugated equine oestrogen; CPRD=Clinical Practice Research Datalink; E2=estradiol; HRT=hormone replacement therapy; MPA=medroxyprogesterone acetate.

* Odds ratios are based on cases and controls matched by age and practice and adjusted for smoking status, body mass index, family history of VTE, chronic and recent medical events, other drugs, and past exposures to hormones.

† Other progestogens include norgestrel/levonorgestrel and drospirenone.

**Table 3:** Direct comparisons between different types of hormone replacement therapy (HRT). Values are adjusted odds ratios (95% confidence intervals)

HRT comparison	QResearch	CPRD	Combined analysis	P
Oral *v* transdermal	1.74 (1.57 to 1.93)	1.61 (1.39 to 1.87)	1.70 (1.56 to 1.85)	<0.001
E2 only *v* CEE only	0.87 (0.76 to 1.00)	0.81 (0.66 to 0.98)	0.85 (0.76 to 0.95)	0.005
E2 combined *v* CEE combined	0.85 (0.76 to 0.94)	0.81 (0.69 to 0.94)	0.83 (0.76 to 0.91)	<0.001
CEE norgestrel *v* CEE MPA	0.72 (0.61 to 0.85)	1.07 (0.85 to 1.35)	0.87 (0.59 to 1.29)	0.5
E2 MPA *v* CEE MPA	0.70 (0.50 to 1.00)	0.64 (0.39 to 1.07)	0.68 (0.51 to 0.91)	0.01
E2 dydrogesterone *v* CEE MPA	0.54 (0.42 to 0.69)	0.61 (0.42 to 0.88)	0.56 (0.45 to 0.69)	<0.001
E2 norethisterone *v* CEE MPA	0.76 (0.66 to 0.87)	0.88 (0.72 to 1.08)	0.80 (0.71 to 0.89)	<0.001
E2 other *v* CEE MPA	0.60 (0.39 to 0.93)	0.88 (0.44 to 1.76)	0.67 (0.47 to 0.97)	0.03

CEE=conjugated equine oestrogen; CPRD=Clinical Practice Research Datalink; E2=estradiol; MPA=medroxyprogesterone acetate.

**Fig 3:**
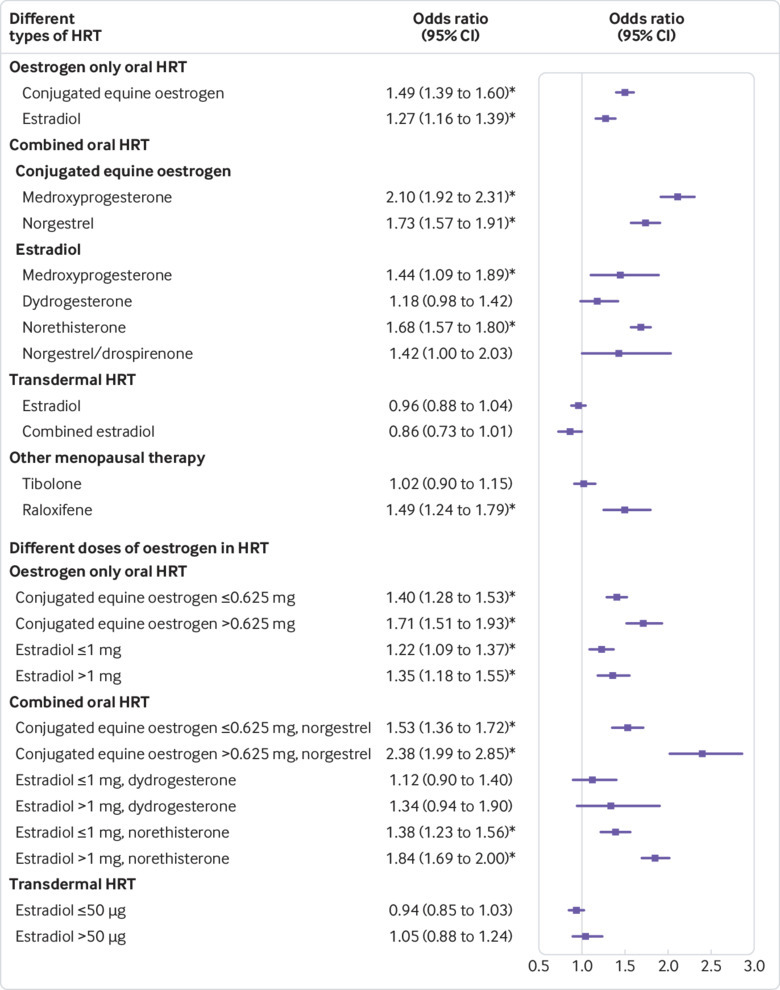
Adjusted odds ratios for different types of hormone replacement therapy (HRT) and different doses of oestrogen. Odds ratios are adjusted for current use of conjugated equine oestrogen cream, estradiol pessaries, oral progestogen, progesterone cream or vaginal preparations, past use of HRT, smoking status, alcohol consumption, Townsend deprivation fifth (QResearch only), body mass index, comorbidities, recent events, current and past use of antidepressants, antipsychotics, aspirin, oral contraceptives, tamoxifen, and years of data. Cases are matched to controls by age, general practice, and index date. *P<0.01

Oral oestrogen only and oral combined preparations were associated with increased VTE risk (adjusted odds ratio 1.40, 95% confidence interval 1.32 to 1.48, and 1.73, 1.65 to 1.81, respectively; table 2). Different types of oestrogen were associated with different risks. Oestrogen only preparations using conjugated equine oestrogen had higher VTE risks than preparations using estradiol (fig 3). Compared with oestrogen only conjugated equine oestrogen, use of oestrogen only estradiol was associated with a 15% reduction in VTE risk (0.85, 0.76 to 0.95; table 3). For combined oral preparations, the risks were significantly increased for conjugated equine oestrogen (1.91, 1.79 to 2.05) and estradiol preparations (1.59, 1.49 to 1.69; table 2) compared with no HRT use in the past year. Direct comparison between the types of oestrogen showed a 17% lower risk for combined estradiol than for combined conjugated equine oestrogen (0.83, 0.76 to 0.91; table 3).

For oral combined HRT, conjugated equine oestrogen with medroxyprogesterone acetate was associated with the highest risk of VTE (adjusted odds ratio 2.10, 95% confidence interval 1.92 to 2.31), and estradiol with dydrogesterone with the lowest risk (1.18, 0.98 to 1.42; table 2 and fig 3). Compared with conjugated equine oestrogen with medroxyprogesterone, estradiol with dydrogesterone and estradiol with norethisterone were associated with 44% and 20% lower VTE risks, respectively (0.56, 0.45 to 0.69, P<0.001, and 0.80, 0.71 to 0.89, P<0.001, respectively; table 3).

Overall, continuous and cyclical regimens for combined oral preparations were associated with an increased risk of VTE compared with no HRT use (adjusted odds ratio 1.55, 95% confidence interval 1.44 to 1.66, and 1.88, 1.77 to 1.99, respectively; table 2). However, not all combinations and age groups were equally covered. Younger women were more likely to be prescribed cyclical preparations and older women continuous preparations (supplementary eTable 3). Not all combined preparations were prescribed for cyclical and continuous regimens; conjugated equine oestrogen with medroxyprogesterone acetate was available mostly as a continuous regimen, whereas conjugated equine oestrogen with norgestrel was generally cyclical (fig 2 and supplementary eFigure 1). Only two preparations, estradiol with dydrogesterone and estradiol with norethisterone, had sufficient observations to assess the effect of the regimen. Neither cyclical nor continuous estradiol with dydrogesterone were associated with a statistically significantly increased VTE risk (1.21, 0.95 to 1.53, and 1.13, 0.84 to 1.53, respectively; table 2). Cyclical and continuous use of estradiol with norethisterone were associated with increased VTE risk compared with no HRT use (1.44, 1.28 to 1.63, and 1.80, 1.66 to 1.95, respectively; table 2).

A large proportion of women using transdermal HRT had oestrogen only preparations (80% (n=781) in cases and 76% (n=3850) in controls (table 2). For combined preparations, norethisterone was the most common progestogen, with very low numbers for levonorgestrel and dydrogesterone, and most of the transdermal preparations had a lower dose of estradiol (fig 2). None of the transdermal preparations (oestrogen only or combined, low dose or high dose, combined cyclical or continuous) was associated with an increased VTE risk (table 2). Only a small proportion of women (about 10%) had a short exposure of fewer than 84 days, and we did not detect any differences in risk compared with longer exposure (supplementary eTable 4).

Tibolone was used by 368 women with VTE and 1859 controls, and its use was not associated with VTE risk (adjusted odds ratio 1.02, 95% confidence interval 0.90 to 1.15; table 2). A small number of women (180 cases and 631 controls) used raloxifene, which was associated with a significantly increased VTE risk (1.49, 1.24 to 1.79; table 2). Use of conjugated equine oestrogen cream or estradiol vaginal preparations was not associated with VTE risk (table 2). Past exposures to HRT in the 91-365 days before the index date were not significantly associated with increased VTE risk (supplementary eTable 5).

### Numbers needed to harm and excess risk of VTE

The rate of VTE for the unexposed population based on the CPRD cohort was 16.0 per 10 000 women years. The rate differed among age groups: 9.0 per 10 000 women years for age 40-54, 22.2 for age 55-64, and 35.1 for age 65-79. Additional VTE cases were expected because of the increased VTE risk for users of most oral preparations (table 4).

**Table 4:** Numbers needed to harm and excess risk of VTE per 10 000 women for different types of HRT over one year

	Numbers needed to harm over one year (95% CI)					Extra VTE cases per 10 000 treated per year (95% CI)			
	All ages	Age 40-54	Age 55-64	Age 64-79		All ages	Age 40-54	Age 55-64	Age 64-79
Oral HRT	1076 (974 to 1196)	2037 (1688 to 2523)	655 (567 to 766)	536 (439 to 674)		9 (8 to 10)	5 (4 to 6)	15 (13 to 18)	19 (15 to 23)
CEE	1273 (1037 to 1613)	1797 (1274 to 2791)	1138 (786 to 1889)	676 (467 to 1117)		8 (6 to 10)	6 (4 to 8)	9 (5 to 13)	15 (9 to 21)
E2	2311 (1610 to 3841)	4011 (2332 to 10 720)	NE	NE		4 (3 to 6)	2 (1 to 4)	NE	NE
CEE MPA	567 (479 to 682)	1199 (783 to 2085)	334 (268 to 426)	268 (198 to 383)		18 (15 to 21)	8 (5 to 13)	30 (23 to 37)	37 (26 to 50)
CEE NG	859 (689 to 1104)	1178 (884 to 1651)	659 (452 to 1076)	382 (233 to 765)		12 (9 to 15)	8 (6 to 11)	15 (9 to 22)	26 (13 to 43)
E2 MPA	1428 (700 to 6779)	NE	NE	NE		7 (1 to 14)	NE	NE	NE
E2 NEA	924 (785 to 1105)	2162 (1535 to 3378)	455 (374 to 565)	485 (336 to 787)		11 (9 to 13)	5 (3 to 7)	22 (18 to 27)	21 (13 to 30)

CEE=conjugated equine oestrogen; E2=estradiol; HRT=hormone replacement therapy; MPA=medroxyprogesterone acetate; NE=no evidence; NEA=norethisterone acetate; NG=norgestrel.

For overall oral HRT use across all age groups, the number needed to harm was 1076 (95% confidence interval 974 to 1196) and the number of extra VTE cases was nine per 10 000 women years (95% confidence interval 8 to 10; table 4). The highest number of extra cases was for conjugated equine oestrogen with medroxyprogesterone acetate (18 per 10 000 women years, 15 to 21), but this also increased with age (8 per 10 000, 5 to 13, for age 40-54; 37 per 10 000, 26 to 50, for age 64-79; table 4).

### Additional analyses

**Fig 4:**
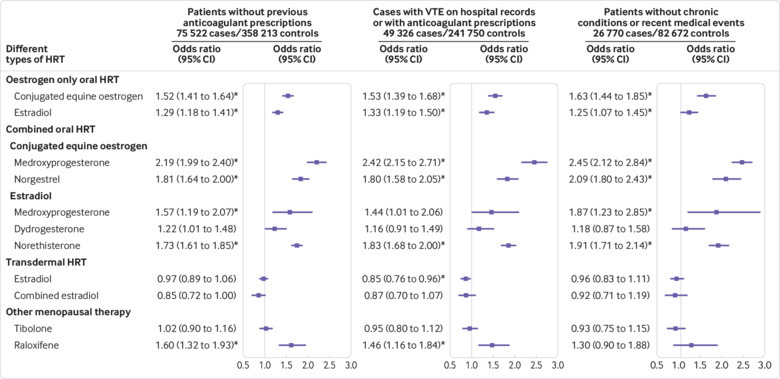
Adjusted odds ratios for different types of hormone replacement therapy (HRT) for additional analyses. Odds ratios are adjusted for current use of conjugated equine oestrogen cream, estradiol pessaries, oral progestogen, progesterone cream or vaginal preparations, past use of HRT, smoking status, alcohol consumption, Townsend deprivation fifth (QResearch only), body mass index, comorbidities, recent events, current and past use of antidepressants, antipsychotics, aspirin, oral contraceptives, tamoxifen, and years of data. Cases are matched to controls by age, general practice, and index date. *P<0.01

The first sensitivity analysis run on cases and controls without previous anticoagulant prescriptions produced similar results to the main analysis, with a consistent difference among odds ratios of up to 0.02. In this analysis, estradiol with medroxyprogesterone acetate was associated with a statistically significantly increased VTE risk (fig 4 and supplementary eTable 6). The second sensitivity analysis used only hospital and mortality linked data for CPRD and produced similar results (supplementary eTable 7). The third sensitivity analysis run on cases and controls with complete data also gave similar findings (supplementary eTable 8). The results from the analysis of cases with associated anticoagulant prescriptions or a VTE diagnosis from hospital or mortality records were similar; however, the reduced risks observed for women using transdermal therapy were statistically significant in the QResearch analysis, and consequently, in the combined analysis (fig 4 and supplementary eTable 9). The subgroups for all these sensitivity analyses had similar HRT exposures to the main groups of cases and controls.

Selected women in the subgroups of idiopathic cases and matched controls had to be free of comorbidities; therefore, these subgroups were younger than the main sample. Women in the subgroups had higher exposure to HRT (9.0% *v* 7.2% for cases and 6.6% *v* 5.5% for controls; table 2 and supplementary eTable 10). The average number of matched controls per case was lower than in the main analysis (mean 3.09 compared with 4.87). Findings were generally similar to the main analysis but with slightly wider confidence intervals. However, preparations with estradiol and medroxyprogesterone acetate were also associated with a statistically significant increased risk in VTE (fig 4 and supplementary eTable 10).

**Fig 5:**
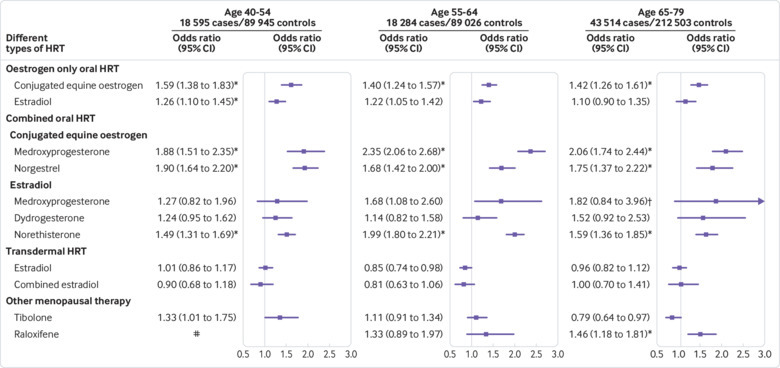
Adjusted odds ratios for different types of hormone replacement therapy (HRT) by age group. Odds ratios are adjusted for current use of conjugated equine oestrogen cream, estradiol pessaries, oral progestogen, progesterone cream or vaginal preparations, past use of HRT, smoking status, alcohol consumption, Townsend deprivation fifth (QResearch only), body mass index, comorbidities, recent events, current and past use of antidepressants, antipsychotics, aspirin, oral contraceptives, tamoxifen, and years of data. Cases are matched to controls by age, general practice, and index date. #=insufficient data. *P<0.01. †Based on QResearch analysis only

Women in the age group 55-64 had the most HRT exposure (13.3% cases and 10.3% controls; supplementary eTable 12) compared with age groups 40-54 (10.8% cases and 7.8% controls; supplementary eTable 11) and 65-79 (3.1% cases and 2.6% controls; supplementary eTable 13). The results were consistent across age subgroups and with the main analysis, but the odds ratios were slightly higher for the age group 55-64. Overall exposure for oral HRT was associated with a significantly increased VTE risk in all age groups (adjusted odds ratio 1.52, 95% confidence interval 1.42 to 1.63, for age 40-54; 1.69, 1.59 to 1.80, for age 55-64; and 1.53, 1.42 to 1.65, for age 65-79; fig 5 and supplementary eTables 11-13).

**Fig 6:**
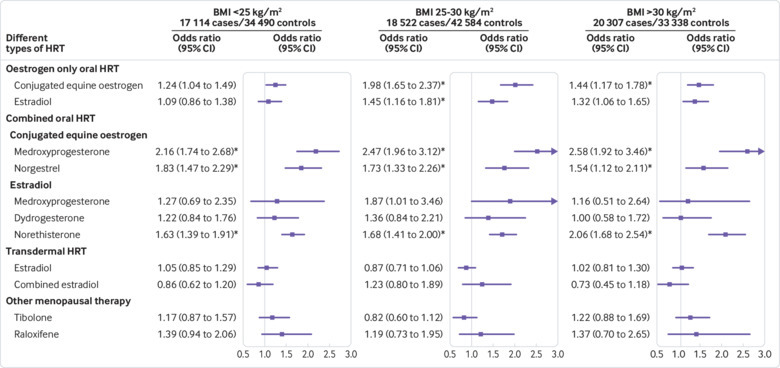
Adjusted odds ratios for different types of hormone replacement therapy (HRT) by body mass index (BMI). Odds ratios are adjusted for current use of conjugated equine oestrogen cream, estradiol pessaries, oral progestogen, progesterone cream or vaginal preparations, past use of HRT, smoking status, alcohol consumption, Townsend deprivation fifth (QResearch only), body mass index, comorbidities, recent events, current and past use of antidepressants, antipsychotics, aspirin, oral contraceptives, tamoxifen, and years of data. Cases are matched to controls by age, general practice, and index date. BMI=body mass index. *P<0.01

Selecting women with VTE in a specific body mass index category and restricting matched controls to women in the same body mass index category resulted in a reduced number of controls per case (not overweight or obese, mean 2.01; overweight, 1.81; obese, 1.64). Women who were not overweight or obese had the highest exposure to HRT (8.8% cases and 7.0% controls; supplementary eTable 14) compared with those who were overweight (7.8% cases and 5.3% controls; supplementary eTable 15) and obese (5.4% cases and 3.8% controls; supplementary eTable 16). The overall risk for oral HRT was the highest in the overweight subgroup (adjusted odds ratio 1.50, 95% confidence interval 1.37 to 1.64, for women who were not overweight or obese; 1.79, 1.63 to 1.97, for women who were overweight; and 1.65, 1.48 to 1.84, for women who were obese; supplementary eTables 14-16). For the different types of HRT, the confidence intervals were wider but the results were consistent among all body mass index subgroups and with the main analysis (fig 6 and supplementary eTables 14-16).

## Discussion

### Statement of principal findings

This study showed increased VTE risks for all oral HRT formulations, including combined and oestrogen only preparations. Overall, preparations with conjugated equine oestrogen were associated with higher risks than preparations using estradiol. Conjugated equine oestrogen with medroxyprogesterone acetate had the highest risk and estradiol with dydrogesterone had the lowest risk. Higher doses of oestrogen were associated with higher VTE risks. Transdermal HRT was not associated with any increased VTE risk and this finding was consistent for different regimens.

### Strengths and weaknesses of the study

This observational study of the UK general female population aged 40-79 used data from the two largest UK primary care databases, and routinely collected primary care data linked to secondary care data, and mortality records. The study matched cases and controls by age and general practice to account for differences in recording and prescribing across practices. Analyses adjusted for many confounding factors such as chronic and acute conditions, lifestyle factors, and social deprivation.

The study included all eligible cases, and additional analyses addressed assumptions. One sensitivity analysis in a subgroup of women with no previous use of anticoagulants reported similar results to the main analysis. This sensitivity analysis indicates that most of the excluded women had probably used anticoagulants because of atrial fibrillation or hip replacement operations rather than an earlier unrecorded VTE.

Records of test results confirming VTE diagnoses were not available to researchers. However, the sensitivity analysis, restricted to cases with subsequent prescriptions for anticoagulants or a VTE diagnosis from hospital or mortality records, supported the findings of the main analysis. In both the main analysis and the additional analysis, use of transdermal treatment was associated with a slight absolute reduction in VTE risk. There is no biological explanation for this protective effect; therefore, this small decrease might reflect some residual confounding or possible indication bias. The finding was not statistically significant in the main analysis, but we reported a slightly greater, statistically significant decrease in the sensitivity analysis for the QResearch data. This is likely a spurious finding, possibly related to selection bias.

The study did not provide information about risks in patients with various conditions, but did separate VTE risks associated with HRT from those associated with chronic and acute conditions by running an additional analysis on patients without these conditions. The results proved similar and suggest a probable independent effect of HRT on VTE risk. Additional analyses based on age and body mass index provided extra information, but the results remained consistent across the categories and with the main results.

Although the analysis included all HRT preparations available in the UK, some preparations were not sufficiently prescribed to be assessed individually, such as estradiol with drospirenone, or estradiol with norgestrel or levonorgestrel. Frequently prescribed preparations, however, did have enough observations to study dosage effects. We were unable to assess differences in risks for women who had recently started or restarted HRT because the majority of women had been using HRT for more than 84 days.

The study also could not address several uncertainties. Exposure information was based on HRT prescriptions and not actual use. However, it is unlikely that cases and controls differed systematically in their adherence to HRT. Although we used all available information on confounding factors, data on important factors such as precise indications for HRT, age at menopause, and education level were not available. For a small but non-negligible proportion of women, data on smoking status, alcohol consumption, and body mass index were missing and had to be multiply imputed for the analysis. All of these limitations could have resulted in some residual confounding bias.

### Comparison with other studies

Several randomised controlled trials have assessed the safety of HRT, but most have not been sufficiently powered to assess VTE outcomes. Seventy per cent of data used in two recent meta-analyses^4^ ^14^ were derived from two arms of one large trial on healthy American women (Women’s Health Initiative).^6^ The trial also concentrated on conjugated equine oestrogen based preparations most commonly used in the US. The Cochrane meta-analysis^4^ included VTE risks for up to two years of exposure to oestrogen only conjugated equine oestrogen (risk ratio 2.22, 95% confidence interval 1.12 to 4.39) and conjugated equine oestrogen with medroxyprogesterone acetate (2.98, 1.88 to 4.71); the findings were slightly higher than those in our study. The other meta-analysis^14^ pooled oestrogen only preparations and combined oestrogen with progestogen preparations and reported increased VTE risks for both types of HRT (relative risk 1.43, 95% confidence interval 1.11 to 1.85; and 1.95, 1.54 to 2.47). These results are similar to our findings for oral preparations.

Most of the observational studies did not distinguish between different types of HRT and assessed the overall risks associated with all formulations. Several studies had separated oral and transdermal use and reported higher risks for oral HRT.^15^ ^16^ ^17^ ^18^ ^19^ One meta-analysis reported the combined findings of oral versus transdermal HRT (relative risk 1.63, 95% confidence interval 1.40 to 1.90).^5^ These results are in line with our findings (odds ratio for oral HRT *v* transdermal HRT 1.70, 95% confidence interval 1.56 to 1.85).

Some studies have assessed the risks associated with different oestrogen doses and reported higher risks with higher doses.^18^ ^19^ ^20^ One study compared cyclical use with continuous use and found higher risks associated with continuous use.^19^ Some studies based on the US population included the most frequently prescribed HRT, conjugated equine oestrogen with medroxyprogesterone, simply extrapolating the results to all synthetic progesterones.^21^ ^22^ A US study extended the exposure to estradiol with medroxyprogesterone, but the study was not sufficiently powered to show increased VTE risk.^20^ Two French studies investigated VTE risks associated with different pharmacological classes of progestogens and found that pregnane progesterones including dydrogesterone and medroxyprogesterone acetate were not associated with increased VTE risk.^16^ ^17^ However, these two studies were insufficiently powered and produced findings with wide confidence intervals.

A UK study, the Million Women Study, conducted between 1997 and 2002 and based on women aged 50-64, included a range of preparations such as oral conjugated equine oestrogen, estradiol, and progestogens medroxyprogesterone, norethisterone, and norgestrel. Results for individual progestogens were, however, reported across both types of oestrogen and for different doses. The results, although less powered (2200 exposed cases), were similar to the findings from our study for oestrogen only HRT (relative risk 1.46, 95% confidence interval 1.23 to 1.75, for conjugated equine oestrogen; and 1.45, 1.06 to 1.98, for estradiol); however, the study reported slightly higher VTE risks associated with combined HRT (2.07, 1.86 to 2.32).^19^

A Cochrane review summarised evidence on the side effects of tibolone and showed no association with VTE^23^; our results were in line with this finding. For increased VTE risk in raloxifene users, our observational study supported the results from a meta-analysis based on 189 exposed cases in randomised controlled trials (odds ratio 1.62, 95% confidence interval 1.25 to 2.09).^24^

### Meaning of the study: possible explanations and implications for clinicians and policy makers

This study has provided a more detailed picture of the VTE risks for different HRT preparations and can help clinicians and women make treatment choices. The study has shown that preparations based on conjugated equine oestrogen are associated with higher VTE risks than estradiol preparations, and this finding is consistent across age and body mass index categories. We did not expect to find an association between transdermal use and VTE risk because of the metabolic process,^25^ which has been confirmed in previous studies.^16^ ^19^ ^26^ However, our study showed that the vast majority of women using HRT continue to be prescribed oral preparations. When women with menopausal symptoms already have an increased VTE risk because of comorbidities or obesity, these women and their doctors should give greater consideration to transdermal HRT, in line with the NICE guideline.^3^

### Unanswered questions and future research

The HRT research recommendations from NICE raise concerns about VTE risk and about some cancers—in particular breast cancer—so a complementary detailed study of cancer risks is needed for a more complete picture. We are preparing such a study, based on the same data sources.

### Conclusion

This large observational study, based on the UK general female population aged 40-79, provides information on VTE risk in women taking different types of HRT. The study shows that conjugated equine oestrogen based oral preparations, combined or oestrogen only, are associated with higher VTE risk than estradiol based preparations. Higher oestrogen dose is also associated with higher VTE risk. However, transdermal HRT or tibolone, used much less frequently, are not associated with any increased VTE risk. Therefore, the study provides more information for clinicians and women about relative VTE risks and highlights that oral HRT is still the preferred choice over other forms of treatment with no associated VTE risks.

**What is already known on this topic**

Randomised controlled trials in women with menopausal symptoms who use hormone replacement therapy have demonstrated increased risks of venous thromboembolism compared with no exposure
The conclusions were based mostly on preparations of conjugated equine oestrogen with and without medroxyprogesterone acetate
Observational studies have reported increased risks associated with overall hormone replacement therapy, but were not sufficiently powered to provide detailed comparisons between different types of treatment

**What this study adds**

This large study, based on routinely collected data from primary care environments, analysed a number of individual types of hormone replacement therapy; most oral preparations were found to be associated with increased venous thromboembolism risks
Conjugated equine oestrogen preparations, with and without medroxyprogesterone acetate, were associated with the highest risks
No increased risk of venous thromboembolism was found for transdermal preparations

## Web extra

Extra material supplied by authors

**Web appendix:** Supplementary material

## Notes

Contributors: YV contributed to the study protocol, reviewed the literature, designed the study, organised the extraction of CPRD data, did the analysis on both datasets, and wrote the draft of the manuscript. JHC initiated the study, undertook the original literature review, drafted the study protocol, organised the extraction of the QResearch data, advised on clinical aspects of the study and interpretation of the results and drafting of the paper. CC contributed to the development of the idea and the study design and advised on the analysis and interpretation of the results. JHC and CC critically reviewed the paper. YV is the guarantor of the study. All authors have approved the submitted version. The corresponding author attests that all listed authors meet authorship criteria and that no others meeting the criteria have been omitted.

Ethical approval: The protocol for QResearch has been published in ePrints and was reviewed in accordance with the requirements for the East Midlands Derby research ethic committee (ref 03/4/021). The protocol for CPRD has been approved by the Independent Scientific Advisory Committee for MHRA Database Research (N 16_275R).

Data sharing: To guarantee the confidentiality of personal and health information only the authors have had access to the data during the study in accordance with the relevant licence agreements. Access to the QResearch data is according to the information on the QResearch website (www.qresearch.org). CPRD linked data were provided under a licence that does not permit sharing.

The lead author affirms that the manuscript is an honest, accurate, and transparent account of the study being reported; that no important aspects of the study have been omitted and that any discrepancies from the study as planned have been explained.
