# Use of hormone replacement therapy and risk of venous thromboembolism: nested case-control studies using the QResearch and CPRD databases

**DOI:** 10.1136/bmj.l162

**Published:** 2019-01-15

This article by Yana Vinogradova and colleagues (*BMJ* 2019;364:k4810, doi:10.1136/bmj.k4810), there were a few text errors, which have since been corrected. These amendments include:

- In the Results section of the abstract, adding a space between “estradiol” and “had”.
- In the second paragraph of the Results, removing the repeat use of “than controls” to read: “Women who had VTE were more likely than controls to have recent medical events (27% *v* 12%), such as respiratory or urinary infection (20% *v* 10%), hip fracture or operation (3.4% *v* 0.3%), or hospital admission (7% *v* 1%), and to use antidepressants (24% *v* 14%; table 1).”
- In the eighth paragraph of the Results, changing the first sentence to begin with: “Overall, cyclical and continuous regimens for combined oral preparations were associated with an increased risk of VTE compared with no HRT use,” to correspond with data in the rest of the sentence.
- In the regimen section of table 2, swapping the data between the cyclical and continuous categories for the first two columns (number of cases/controls and adjusted odds ratios (95% confidence intervals)), to read:
  - Combined cyclical: 787\3164; 1.48 (1.36 to 1.62)
  - Combined continuous: 1123\3606; 1.96 (1.82 to 2.11)
  - EE Dydrogesterone cyclical: 63\312; 1.23 (0.92 to 1.64)
  - EE Dydrogesterone continuous: 38\208; 1.13 (0.78 to 1.63)
  - EE Norethisterone cyclical: 263\1080; 1.42 (1.23 to 1.65)
  - EE Norethisterone continuous: 585\1963; 1.85 (1.67 to 2.04).

