# Supplementary material for: Use of hormone replacement therapy and risk of venous thromboembolism: nested case-control studies using the QResearch and CPRD databases
Source: BMJ. 2019 Jan 9;364:k4810. doi: 10.1136/bmj.k4810 (PMC6326068; doi:10.1136/bmj.k4810)
Supplement: Supplementary file 1 — Web appendix: Supplementary material [file viny046401.ww.pdf]

Use of hormone replacement therapy and risk of venous thromboembolism: two nested case-control studies in primary care

Supplementary tables

eTable 1 Characteristics in cases and controls at or before the index date across the databases by exposure to HRT 3
eTable 2 **All cases and controls**: Odds ratios for age-practice matched cases and controls for any HRT exposure. 5
eTable 3 Proportion (number) of cases and controls prescribed different regimens of oral HRT, across both databases 6
eTable 4 **All cases and controls:** Duration of use for different types of HRT with sufficient number of observation by database 7
eTable 5 **All cases and controls:** Past use (91-365 days before the index date) of different oestrogen and progestogens, number of cases and controls and adjusted odds ratios by database and combined analysis 8
eTable 6 **Cases and controls without previous anticoagulant prescription:** Numbers of cases and controls and adjusted odds ratios by database and combined odds ratios 9
eTable 7 **Cases and controls with data linked to hospital and mortality data:** Numbers of cases and controls and adjusted odds ratios by database and combined odds ratios 10
eTable 8 **Cases and controls with complete data for body-mass index, smoking status and alcohol consumption:** Numbers of cases and controls and adjusted odds ratios by database and combined odds ratios 11
eTable 9 **Cases restricted to patients with a diagnosis of VTE on hospital or mortality records, or with associated anticoagulant prescriptions, and their controls:** Numbers of cases and controls and adjusted odds ratios by database and combined odds ratios 12
eTable 10 **Cases and controls without chronic conditions or recent medical events:** Numbers of cases and controls and adjusted odds ratios by database and combined odds ratios 13
eTable 11 **Cases and controls aged 40 to 54:** Numbers of cases and controls and adjusted odds ratios by database and combined odds ratios 14
eTable 12 **Cases and controls aged 55 to 64:** Numbers of cases and controls and adjusted odds ratios by database and combined odds ratios 15
eTable 13 **Cases and controls aged 65 to 79:** Numbers of cases and controls and adjusted odds ratios by database and combined odds ratios 16
eTable 14 **Cases and controls with body mass index < 25kg/m²:** Numbers of cases and controls and adjusted odds ratios by database and combined odds ratios 17
eTable 15 **Cases and controls with body mass index 25 to 30kg/m²:** Numbers of cases and controls and adjusted odds ratios by database and combined odds ratios 18
eTable 16 **Cases and controls with body mass index > 30kg/m²:** Numbers of cases and controls and adjusted odds ratios by database and combined odds ratios 19

eFigure 1 HRT preparations available in the UK and the number of controls from both databases

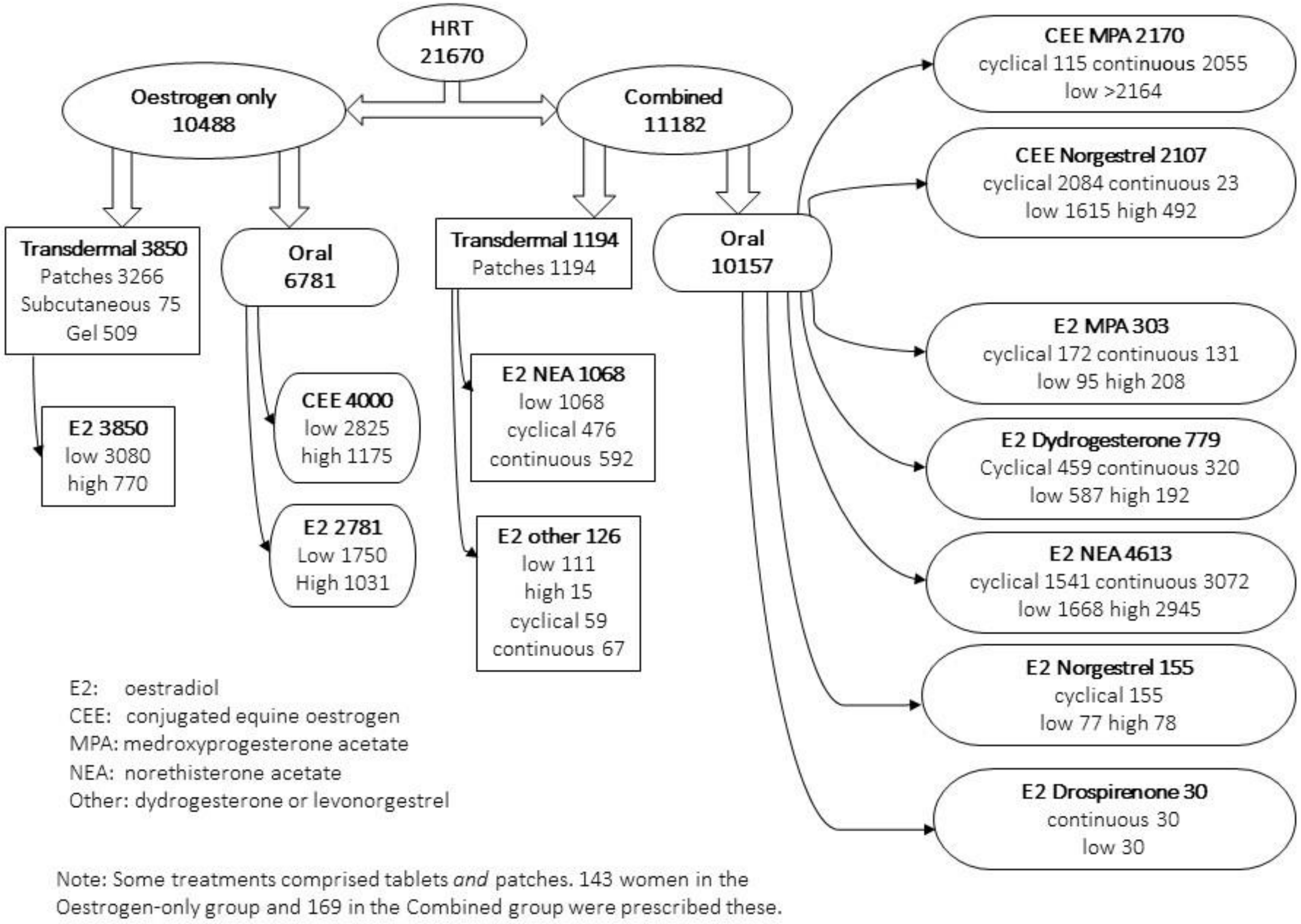

eTable 1 Characteristics in cases and controls at or before the index date across the databases by exposure to HRT

	No HRT		Oral HRT		Transdermal HRT	
	Cases; % (N)	Controls; % (N)	Cases; % (N)	Controls; % (N)	Cases; % (N)	Controls; % (N)
Total	74601	369824	4915	16938	982	5044
**Age in years**						
40 to 54	22.2 (16585)	22.4 (82899)	34.8 (1711)	33.3 (5639)	36.6 (359)	31.2 (1572)
55 to 64	21.3 (15861)	21.6 (79848)	42.6 (2094)	42.8 (7244)	36.5 (358)	40.9 (2064)
65 to 79	56.5 (42155)	56.0 (207077)	22.6 (1110)	23.9 (4055)	27.0 (265)	27.9 (1408)
**Ethnic group: non-white**	4.4 (3257)	4.8 (17771)	1.8 (89)	2.0 (344)	1.8 (18)	2.1 (107)
**Body mass index**						
15 to 24 kg/m^2	23.8 (17752)	33.0 (122106)	29.1 (1432)	41.7 (7069)	30.3 (298)	39.2 (1976)
25 to 29 kg/m^2	28.0 (20859)	29.8 (110278)	30.8 (1515)	29.8 (5055)	30.4 (299)	31.7 (1600)
30 kg/m^2 and over	35.9 (26748)	23.3 (86206)	28.8 (1417)	16.6 (2819)	28.2 (277)	17.6 (889)
not recorded	9.5 (7110)	10.2 (37642)	8.0 (395)	8.9 (1507)	8.5 (83)	8.9 (449)
**Smoking**						
none	46.7 (34853)	49.7 (183961)	43.6 (2142)	45.0 (7621)	43.3 (425)	47.1 (2374)
ex	32.6 (24311)	29.6 (109349)	30.1 (1479)	29.1 (4924)	30.4 (299)	31.1 (1567)
light	8.1 (6049)	7.4 (27494)	9.5 (467)	9.6 (1622)	10.2 (100)	8.5 (430)
moderate	4.7 (3469)	4.6 (16891)	5.8 (286)	6.3 (1073)	6.1 (60)	5.5 (277)
heavy	3.3 (2431)	2.7 (9824)	5.9 (289)	4.9 (837)	4.6 (45)	3.5 (177)
not recorded	4.7 (3488)	6.0 (22305)	5.1 (252)	5.1 (861)	5.4 (53)	4.3 (219)
**Alcohol**						
none	29.9 (22278)	26.3 (97156)	24.1 (1186)	21.0 (3561)	24.6 (242)	20.7 (1044)
ex-use	9.2 (6879)	7.8 (28690)	6.8 (333)	5.7 (961)	7.1 (70)	5.5 (278)
trivial	30.5 (22730)	31.9 (118022)	35.3 (1735)	35.6 (6025)	34.9 (343)	36.3 (1832)
light	10.2 (7597)	12.2 (45113)	12.4 (609)	15.1 (2564)	12.4 (122)	16.0 (808)

moderate	5.4 (4051)	6.3 (23248)	7.4 (365)	8.2 (1381)	8.0 (79)	7.6 (381)
heavy	0.6 (421)	0.5 (1702)	0.4 (21)	0.5 (80)	0.7 (7)	0.4 (20)
very heavy	0.4 (285)	0.2 (889)	0.3 (17)	0.2 (33)	0.3 (3)	0.2 (9)
not recorded	13.9 (10360)	14.9 (55004)	13.2 (649)	13.8 (2333)	11.8 (116)	13.3 (672)
**Morbidities**	57.1 (42617)	36.1 (133327)	45.4 (2232)	29.5 (5003)	52.1 (512)	33.0 (1663)
asthma	15.6 (11667)	11.3 (41935)	18.6 (912)	13.2 (2230)	18.4 (181)	13.8 (697)
atrial fibrillation	3.6 (2675)	2.6 (9790)	1.5 (75)	1.0 (173)	2.2 (22)	1.3 (68)
cancer	21.7 (16196)	7.0 (25792)	9.9 (486)	3.0 (503)	14.4 (141)	4.0 (200)
cardiovascular disease	13.6 (10147)	9.4 (34857)	8.0 (391)	4.9 (826)	10.3 (101)	5.8 (293)
COPD	7.1 (5280)	3.9 (14304)	5.1 (253)	2.7 (457)	4.3 (42)	2.5 (127)
chronic renal disease	8.9 (6605)	5.7 (20987)	3.3 (162)	1.6 (279)	4.3 (42)	2.0 (102)
congestion cardiac failure	3.1 (2341)	1.5 (5482)	1.5 (75)	0.5 (86)	1.5 (15)	0.6 (29)
Inflammatory bowel disease	1.7 (1282)	1.0 (3551)	1.8 (90)	1.0 (175)	2.3 (23)	1.2 (61)
rheumatoid arthritis	3.8 (2805)	2.0 (7400)	4.2 (207)	2.0 (337)	4.1 (40)	2.3 (118)
SLE	0.4 (307)	0.2 (617)	0.5 (23)	0.2 (29)	1.0 (10)	0.5 (24)
varicose veins	7.2 (5345)	5.2 (19114)	7.4 (363)	6.4 (1081)	10.7 (105)	7.4 (374)
**Recent events**	26.7 (19906)	11.4 (42010)	24.6 (1210)	13.2 (2237)	26.7 (262)	13.8 (697)
respiratory infection	12.7 (9497)	6.5 (24206)	15.6 (766)	9.1 (1546)	17.1 (168)	8.6 (436)
urinary infection	7.0 (5222)	3.7 (13541)	5.5 (271)	3.4 (582)	5.5 (54)	4.5 (229)
**Other characteristics**						
oophorectomy/hysterectomy	27.5 (20482)	22.7 (84098)	44.5 (2185)	43.0 (7278)	72.7 (714)	67.5 (3407)
**Other medications in the last 90 days**						
aspirin	14.1 (10497)	11.3 (41607)	8.0 (391)	6.1 (1036)	9.0 (88)	6.6 (333)
antidepressants	23.1 (17268)	13.5 (50050)	29.7 (1461)	19.5 (3298)	30.0 (295)	20.7 (1045)
antipsychotics	3.4 (2531)	1.1 (4186)	2.7 (133)	1.0 (170)	3.3 (32)	1.0 (49)

eTable 2 ***All cases and controls****: Odds ratios for age-practice matched cases and controls for any HRT exposure.*

	QResearch	CPRD	Combined analysis	
	Unadjusted Odds ratio (95%CI)	Unadjusted Odds ratio (95%CI)	Combined unadjusted Odds ratio (95%CI)	p-value
**ANY HRT**	1.35 (1.30 to 1.40)	1.37 (1.30 to 1.44)	1.36 (1.31 to 1.40)	<0.001
**ORAL PREPARATIONS**	1.48 (1.42 to 1.54)	1.45 (1.37 to 1.54)	1.47 (1.42 to 1.52)	<0.001
**Oestrogen-only**	1.47 (1.38 to 1.57)	1.46 (1.33 to 1.59)	1.47 (1.39 to 1.54)	<0.001
CEE	1.52 (1.40 to 1.65)	1.53 (1.37 to 1.71)	1.53 (1.43 to 1.63)	<0.001
E2	1.40 (1.27 to 1.55)	1.35 (1.17 to 1.55)	1.38 (1.27 to 1.50)	<0.001
**Combined with progestogen**	1.49 (1.41 to 1.57)	1.45 (1.35 to 1.57)	1.48 (1.41 to 1.54)	<0.001
**CEE combined**	1.63 (1.51 to 1.76)	1.59 (1.43 to 1.78)	1.62 (1.52 to 1.72)	<0.001
CEE MPA	1.84 (1.65 to 2.04)	1.55 (1.33 to 1.80)	1.74 (1.59 to 1.89)	<0.001
CEE Norgestrel	1.42 (1.27 to 1.59)	1.64 (1.41 to 1.91)	1.50 (1.37 to 1.64)	<0.001
**E2 combined**	1.39 (1.29 to 1.49)	1.35 (1.22 to 1.49)	1.37 (1.30 to 1.46)	<0.001
E2 MPA	1.29 (0.94 to 1.77)	1.10 (0.70 to 1.72)	1.22 (0.95 to 1.58)	0.1
E2 Dydrogesterone	1.01 (0.81 to 1.25)	0.95 (0.70 to 1.29)	0.99 (0.83 to 1.18)	0.9
E2 Norethisterone	1.47 (1.36 to 1.59)	1.44 (1.29 to 1.60)	1.46 (1.37 to 1.56)	<0.001
E2 other progestogens†	1.08 (0.72 to 1.61)	1.31 (0.70 to 2.45)	1.14 (0.81 to 1.60)	0.4
**Regimen**				
Combined cyclical	1.32 (1.21 to 1.43)	1.45 (1.30 to 1.63)	1.36 (1.27 to 1.46)	<0.001
Combined continuous	1.63 (1.52 to 1.75)	1.45 (1.32 to 1.60)	1.57 (1.48 to 1.66)	<0.001
E2 Dydrogesterone cyclical	1.05 (0.80 to 1.39)	1.02 (0.69 to 1.51)	1.04 (0.83 to 1.30)	0.7
E2 Dydrogesterone continuous	0.93 (0.66 to 1.32)	0.86 (0.53 to 1.39)	0.91 (0.68 to 1.20)	0.5
E2 Norethisterone cyclical	1.30 (1.13 to 1.49)	1.38 (1.13 to 1.68)	1.32 (1.18 to 1.48)	<0.001
E2 Norethisterone continuous	1.57 (1.43 to 1.72)	1.46 (1.29 to 1.65)	1.53 (1.42 to 1.65)	<0.001
**Oral oestrogen dose**				
CEE ≤0.625mg	1.34 (1.21 to 1.48)	1.46 (1.27 to 1.68)	1.38 (1.27 to 1.50)	<0.001
CEE >0.625mg	2.01 (1.75 to 2.31)	1.69 (1.40 to 2.04)	1.89 (1.69 to 2.11)	<0.001
E2 ≤1mg	1.30 (1.14 to 1.48)	1.30 (1.09 to 1.57)	1.30 (1.17 to 1.44)	<0.001
E2 >1mg	1.58 (1.35 to 1.85)	1.41 (1.14 to 1.76)	1.52 (1.34 to 1.73)	<0.001
CEE ≤0.625mg Norgestrel	1.28 (1.11 to 1.46)	1.36 (1.13 to 1.63)	1.30 (1.17 to 1.46)	<0.001
CEE >0.625mg Norgestrel	1.92 (1.56 to 2.37)	2.61 (1.99 to 3.41)	2.16 (1.83 to 2.55)	<0.001
E2 ≤1mg Dydrogesterone	0.93 (0.72 to 1.21)	0.88 (0.62 to 1.25)	0.91 (0.74 to 1.13)	0.4

E2 >1mg Dydrogesterone	1.21 (0.82 to 1.78)	1.22 (0.68 to 2.20)	1.21 (0.87 to 1.68)	0.2
E2 ≤1mg Norethisterone	1.15 (1.00 to 1.32)	1.20 (1.00 to 1.44)	1.17 (1.04 to 1.31)	0.008
E2 >1mg Norethisterone	1.66 (1.51 to 1.82)	1.58 (1.38 to 1.79)	1.63 (1.51 to 1.76)	<0.001
**TRANSDERMAL PREPARATIONS**	0.90 (0.83 to 0.98)	1.04 (0.93 to 1.18)	0.95 (0.88 to 1.02)	0.1
E2 only	0.95 (0.87 to 1.05)	1.08 (0.94 to 1.23)	1.00 (0.92 to 1.08)	0.9
E2 combined	0.76 (0.63 to 0.91)	0.94 (0.71 to 1.23)	0.81 (0.70 to 0.94)	0.006
combined cyclical	0.77 (0.59 to 1.00)	0.81 (0.52 to 1.29)	0.78 (0.62 to 0.98)	0.03
combined continuous	0.75 (0.59 to 0.97)	1.00 (0.71 to 1.40)	0.83 (0.68 to 1.02)	0.08
**Transdermal oestrogen dose**				
E2 ≤50mcg	0.90 (0.81 to 1.00)	1.02 (0.88 to 1.19)	0.94 (0.86 to 1.03)	0.2
E2 >50mcg	1.18 (0.97 to 1.44)	1.32 (1.00 to 1.74)	1.23 (1.05 to 1.44)	0.01
**OTHER MENOPAUSAL TREATMENT**				
Tibolone	0.95 (0.82 to 1.10)	1.06 (0.88 to 1.27)	0.99 (0.89 to 1.11)	0.9
Raloxifene	1.38 (1.12 to 1.70)	1.51 (1.14 to 1.99)	1.42 (1.20 to 1.68)	<0.001
CEE cream	0.85 (0.54 to 1.34)	1.08 (0.73 to 1.60)	0.97 (0.72 to 1.31)	0.9
E2 vaginal	0.80 (0.64 to 1.01)	0.85 (0.73 to 0.99)	0.84 (0.74 to 0.95)	0.006

†other progestogens include norgestrel/levonorgestrel and drospirenone

eTable 3 Proportion (number) of cases and controls prescribed different regimens of oral HRT, across both databases

	Oestrogen only		Combined cyclical		Combined continuous	
age group	Cases	Controls	Cases	Controls	Cases	Controls
40-54	4.2 (783)	2.5 (2292)	3.7 (686)	2.8 (2518)	1.8 (340)	1.3 (1149)
55-64	4.8 (870)	3.7 (3311)	2.1 (384)	1.7 (1512)	5.7 (1045)	3.8 (3367)
65-79	1.5 (664)	1.3 (2815)	0.3 (128)	0.2 (496)	1.3 (563)	0.9 (1968)

*eTable 4 **All cases and controls:** Duration of use for different types of HRT with sufficient number of observation by database*

	QResearch		CPRD		Combined analysis	
	N of cases/ controls	Adjusted odds ratio (95% confidence interval)	N of cases/ controls	Adjusted odds ratio (95% confidence interval)	Combined odds ratio (95% confidence interval)	P-value
**Oral preparations**						
CEE <=84ds	79 / 215	1.87 (1.41 to 2.47)	36 / 90	1.98 (1.30 to 3.02)	1.90 (1.51 to 2.40)	<0.001
CEE >84ds	707 / 2477	1.47 (1.34 to 1.61)	386 / 1218	1.44 (1.27 to 1.64)	1.46 (1.35 to 1.57)	<0.001
CEE MPA <=84ds	40 / 172	1.44 (1.00 to 2.08)	21 / 61	1.79 (1.03 to 3.10)	1.54 (1.13 to 2.09)	0.006
CEE MPA >84ds	461 / 1266	2.33 (2.07 to 2.61)	218 / 671	1.87 (1.58 to 2.22)	2.17 (1.97 to 2.39)	<0.001
CEE Norgestrel <=84ds	43 / 130	1.91 (1.33 to 2.76)	23 / 53	2.37 (1.40 to 4.01)	2.05 (1.52 to 2.77)	<0.001
CEE Norgestrel >84ds	339 / 1302	1.56 (1.37 to 1.77)	209 / 622	1.97 (1.66 to 2.34)	1.69 (1.53 to 1.88)	<0.001
E2 <=84ds	56 / 212	1.13 (0.82 to 1.55)	29 / 78	1.64 (1.02 to 2.65)	1.27 (0.97 to 1.65)	0.08
E2 >84ds	455 / 1664	1.33 (1.19 to 1.49)	231 / 827	1.16 (0.98 to 1.36)	1.27 (1.16 to 1.40)	<0.001
E2 NEA <=84ds	96 / 339	1.59 (1.24 to 2.03)	49 / 181	1.33 (0.93 to 1.91)	1.50 (1.23 to 1.84)	<0.001
E2 NEA >84ds	752 / 2704	1.71 (1.56 to 1.86)	418 / 1389	1.69 (1.49 to 1.92)	1.70 (1.58 to 1.83)	<0.001
**Transdermal preparations**						
Oestrogen only <=84ds	41 / 218	0.82 (0.57 to 1.17)	55 / 261	1.04 (0.76 to 1.43)	0.94 (0.74 to 1.19)	0.6
Oestrogen only >84ds	462 / 2427	0.95 (0.86 to 1.06)	223 / 943	0.98 (0.83 to 1.15)	0.96 (0.88 to 1.05)	0.4
Combined <=84ds	25 / 91	1.40 (0.86 to 2.26)	11 / 72	0.70 (0.35 to 1.40)	1.11 (0.75 to 1.65)	0.6
Combined >84ds	112 / 782	0.77 (0.62 to 0.95)	53 / 249	0.97 (0.69 to 1.36)	0.82 (0.69 to 0.98)	0.03

CEE conjugated equine oestrogen; E2 oestradiol; MPA medroxyprogesterone acetate, NEA norethisterone acetate

*Odds ratios are based on cases and controls matched by age and practice and adjusted for smoking status, body mass index, family history of VTE, chronic and recent medical events, other medications and past exposures to hormones.

eTable 5 ***All cases and controls:*** *Past use (91-365 days before the index date) of different oestrogen and progestogens, number of cases and controls and adjusted odds ratios by database and combined analysis*

	QResearch		CPRD		Combined analysis	
	N of cases/ controls	Adjusted odds ratio (95% confidence interval)	N of cases/ controls	Adjusted odds ratio (95% confidence interval)	Combined odds ratio (95% confidence interval)	P-value
Oral CEE	309 / 1161	1.17 (0.96 to 1.44)	173 / 546	1.25 (0.94 to 1.65)	1.20 (1.02 to 1.41)	0.03
Oral E2	299 / 1294	1.04 (0.83 to 1.31)	190 / 635	1.24 (0.90 to 1.69)	1.11 (0.92 to 1.33)	0.3
Oral MPA	109 / 419	1.04 (0.78 to 1.39)	56 / 202	0.92 (0.61 to 1.39)	1.00 (0.79 to 1.27)	1
Oral Norgestrel	101 / 407	0.87 (0.64 to 1.18)	59 / 185	0.99 (0.64 to 1.52)	0.91 (0.71 to 1.16)	0.4
Oral Norethisterone	162 / 698	1.13 (0.85 to 1.52)	101 / 357	1.00 (0.68 to 1.49)	1.09 (0.86 to 1.37)	0.5
Oral other progestogens	39 / 186	0.89 (0.59 to 1.36)	24 / 84	1.15 (0.65 to 2.04)	0.97 (0.69 to 1.37)	0.9
Transdermal E2	118 / 414	1.40 (1.07 to 1.84)	107 / 412	0.89 (0.67 to 1.18)	1.13 (0.93 to 1.37)	0.2
Transdermal progestogens	38 / 158	0.79 (0.49 to 1.26)	31 / 94	1.76 (1.03 to 3.00)	1.12 (0.79 to 1.60)	0.5
Raloxifene	22 / 69	1.39 (0.82 to 2.37)	7 / 29	0.98 (0.38 to 2.53)	1.28 (0.80 to 2.04)	0.3
Tibolone	67 / 296	0.98 (0.73 to 1.30)	50 / 154	1.31 (0.91 to 1.89)	1.09 (0.87 to 1.37)	0.4
CEE cream	15 / 95	0.86 (0.49 to 1.51)	11 / 68	0.62 (0.31 to 1.23)	0.75 (0.48 to 1.16)	0.2
E2 vaginal pessaries	65 / 289	1.07 (0.80 to 1.43)	117 / 449	1.17 (0.92 to 1.48)	1.13 (0.94 to 1.35)	0.2

CEE conjugated equine oestrogen; E2 oestradiol; MPA medroxyprogesterone acetate, NEA norethisterone acetate

*Odds ratios are based on cases and controls matched by age and practice and adjusted for smoking status, body mass index, family history of VTE, chronic and recent medical events, other medications and current exposures to hormones.

eTable 6 ***Cases and controls without previous anticoagulant prescription:*** *Numbers of cases and controls and adjusted odds ratios by database and combined odds ratios*

	QResearch		CPRD		Combined analysis	
	N of cases/ controls	Adjusted Odds ratio (95%CI)	N of cases/ controls	Adjusted Odds ratio (95%CI)		
Total N	48765 \ 236295		26757 \ 121918			
**Any HRT**	3647 \ 13612	1.48 (1.41 to 1.54)	1960 \ 6667	1.46 (1.37 to 1.55)	1.47 (1.42 to 1.53)	<0.001
**Oral preparations**	3114 \ 10591	1.64 (1.57 to 1.72)	1659 \ 5293	1.59 (1.49 to 1.70)	1.63 (1.57 to 1.69)	<0.001
**Oestrogen-only**						
CEE	758 \ 2509	1.54 (1.40 to 1.68)	406 \ 1236	1.48 (1.30 to 1.68)	1.52 (1.41 to 1.64)	<0.001
E2	492 \ 1754	1.33 (1.19 to 1.49)	252 \ 865	1.21 (1.03 to 1.42)	1.29 (1.18 to 1.41)	<0.001
**Combined with progestogen**						
CEE MPA	484 \ 1344	2.30 (2.06 to 2.58)	232 \ 688	1.95 (1.65 to 2.30)	2.19 (1.99 to 2.40)	<0.001
CEE Norgestrel	378 \ 1346	1.68 (1.48 to 1.90)	227 \ 624	2.10 (1.77 to 2.48)	1.81 (1.64 to 2.00)	<0.001
E2 MPA	49 \ 184	1.72 (1.23 to 2.41)	24 \ 100	1.28 (0.79 to 2.10)	1.57 (1.19 to 2.07)	0.002
E2 Dydrogesterone	98 \ 490	1.24 (0.98 to 1.56)	51 \ 245	1.19 (0.85 to 1.67)	1.22 (1.01 to 1.48)	0.04
E2 Norethisterone	826 \ 2830	1.75 (1.61 to 1.91)	454 \ 1490	1.68 (1.48 to 1.89)	1.73 (1.61 to 1.85)	<0.001
E2 other progestogens†	29 \ 134	1.38 (0.90 to 2.10)	13 \ 45	1.69 (0.86 to 3.29)	1.46 (1.02 to 2.09)	0.04
**Transdermal preparations**	606 \ 3264	0.92 (0.84 to 1.01)	323 \ 1432	0.98 (0.86 to 1.12)	0.94 (0.87 to 1.02)	0.1
E2 only	476 \ 2455	0.95 (0.85 to 1.06)	264 \ 1128	1.01 (0.86 to 1.17)	0.97 (0.89 to 1.06)	0.5
E2 combined	130 \ 809	0.83 (0.68 to 1.01)	59 \ 304	0.90 (0.66 to 1.23)	0.85 (0.72 to 1.00)	0.06
**Other oral menopausal treatment**						
Tibolone	208 \ 1138	0.98 (0.84 to 1.15)	140 \ 596	1.08 (0.88 to 1.34)	1.02 (0.90 to 1.16)	0.8
Raloxifene	112 \ 380	1.58 (1.25 to 1.99)	65 \ 187	1.64 (1.18 to 2.27)	1.60 (1.32 to 1.93)	<0.001

CEE conjugated equine oestrogen; E2 oestradiol; MPA medroxyprogesterone acetate

*Odds ratios are based on cases and controls matched by age and practice and adjusted for smoking status, body mass index, family history of VTE, chronic and recent medical events, other medications and past exposures to hormones.

†other progestogens include norgestrel/levonorgestrel and drospirenone

eTable 7 ***Cases and controls with data linked to hospital, mortality and deprivation data:*** *Numbers of cases and controls and adjusted odds ratios by database and combined odds ratios*

	**QResearch**		**CPRD**		**Combined analysis**	
	N of cases/ controls	Adjusted Odds ratio (95%CI)	N of cases/ controls	Adjusted Odds ratio (95%CI)		
Total N	52137 / 259542		16638 / 76114			
**Any HRT**	3769 / 14604	1.43 (1.37 to 1.50)	1280 / 4316	1.49 (1.38 to 1.61)	1.45 (1.39 to 1.50)	<0.001
**Oral preparations**	3207 / 11338	1.59 (1.52 to 1.67)	1071 / 3468	1.58 (1.45 to 1.72)	1.59 (1.53 to 1.66)	<0.001
**Oestrogen-only**						
CEE	786 / 2692	1.50 (1.37 to 1.64)	277 / 845	1.53 (1.31 to 1.79)	1.51 (1.39 to 1.63)	<0.001
E2	511 / 1876	1.31 (1.17 to 1.46)	164 / 549	1.29 (1.06 to 1.57)	1.30 (1.19 to 1.43)	<0.001
**Combined with progestogen**						
CEE MPA	501 / 1438	2.22 (1.99 to 2.48)	145 / 428	1.99 (1.61 to 2.46)	2.17 (1.97 to 2.39)	<0.001
CEE Norgestrel	382 / 1432	1.59 (1.41 to 1.80)	142 / 449	1.80 (1.46 to 2.21)	1.64 (1.48 to 1.83)	<0.001
E2 MPA	49 / 197	1.56 (1.12 to 2.18)	16 / 66	1.31 (0.72 to 2.40)	1.50 (1.12 to 2.01)	0.006
E2 Dydrogesterone	101 / 520	1.19 (0.95 to 1.50)	31 / 159	1.05 (0.68 to 1.60)	1.16 (0.95 to 1.42)	0.2
E2 Norethisterone	848 / 3043	1.69 (1.56 to 1.84)	286 / 945	1.64 (1.41 to 1.91)	1.68 (1.56 to 1.81)	<0.001
E2 other progestogens†	29 / 140	1.34 (0.88 to 2.04)	10 / 27	2.23 (1.00 to 4.97)	1.49 (1.03 to 2.17)	0.03
**Transdermal preparations**	640 / 3519	0.92 (0.84 to 1.00)	227 / 876	1.14 (0.97 to 1.35)	0.96 (0.89 to 1.04)	0.4
E2 only	503 / 2646	0.94 (0.85 to 1.04)	184 / 701	1.13 (0.94 to 1.36)	0.98 (0.90 to 1.08)	0.7
E2 combined	137 / 873	0.84 (0.69 to 1.01)	43 / 175	1.20 (0.83 to 1.75)	0.90 (0.76 to 1.07)	0.2
**Other oral menopausal treatment**						
Tibolone	224 / 1218	0.99 (0.85 to 1.16)	92 / 396	1.08 (0.83 to 1.39)	1.01 (0.89 to 1.16)	0.8
Raloxifene	114 / 419	1.48 (1.18 to 1.86)	47 / 116	1.80 (1.22 to 2.67)	1.56 (1.28 to 1.89)	<0.001

CEE conjugated equine oestrogen; E2 oestradiol; MPA medroxyprogesterone acetate

*Odds ratios are based on cases and controls matched by age and practice and adjusted for smoking status, body mass index, Townsend deprivation fifths, family history of VTE, chronic and recent medical events, other medications and past exposures to hormones.

†other progestogens include norgestrel/levonorgestrel and drospirenone

eTable 8 ***Cases and controls with complete data for body-mass index, smoking status and alcohol consumption:*** *Numbers of cases and controls and adjusted odds ratios by database and combined odds ratios*

	QResearch		CPRD		Combined analysis	
	N of cases/ controls	Adjusted Odds ratio (95%CI)	N of cases/ controls	Adjusted Odds ratio (95%CI)		
Total N	41696 / 174639		23306 / 92504			
**Any HRT**	2999 / 9461	1.42 (1.35 to 1.49)	1721 / 4927	1.50 (1.40 to 1.61)	1.45 (1.39 to 1.50)	<0.001
**Oral preparations**	2555 / 7299	1.59 (1.50 to 1.68)	1461 / 3878	1.67 (1.55 to 1.79)	1.61 (1.55 to 1.69)	<0.001
**Oestrogen-only**						
CEE	626 / 1708	1.52 (1.37 to 1.68)	358 / 872	1.63 (1.41 to 1.88)	1.56 (1.43 to 1.69)	<0.001
E2	424 / 1270	1.29 (1.14 to 1.46)	221 / 630	1.29 (1.08 to 1.54)	1.29 (1.16 to 1.43)	<0.001
**Combined with progestogen**						
CEE MPA	390 / 899	2.25 (1.98 to 2.57)	209 / 516	2.01 (1.67 to 2.41)	2.17 (1.95 to 2.41)	<0.001
CEE Norgestrel	276 / 864	1.52 (1.31 to 1.77)	197 / 451	2.12 (1.76 to 2.57)	1.79 (1.29 to 2.48)	<0.001
E2 MPA	43 / 132	1.69 (1.17 to 2.45)	17 / 73	1.17 (0.67 to 2.06)	1.52 (1.11 to 2.07)	0.008
E2 Dydrogesterone	81 / 352	1.15 (0.89 to 1.50)	44 / 188	1.23 (0.85 to 1.76)	1.18 (0.95 to 1.46)	0.1
E2 Norethisterone	692 / 1990	1.69 (1.54 to 1.87)	403 / 1118	1.71 (1.50 to 1.96)	1.70 (1.57 to 1.84)	<0.001
E2 other progestogens†	23 / 84	1.39 (0.85 to 2.26)	12 / 30	1.93 (0.93 to 3.99)	1.53 (1.02 to 2.30)	0.04
**Transdermal preparations**	510 / 2312	0.89 (0.80 to 0.99)	282 / 1090	0.95 (0.82 to 1.11)	0.91 (0.84 to 1.00)	0.04
E2 only	398 / 1750	0.91 (0.81 to 1.03)	226 / 857	0.98 (0.83 to 1.16)	0.93 (0.85 to 1.03)	0.2
E2 combined	112 / 562	0.83 (0.67 to 1.04)	56 / 233	0.86 (0.62 to 1.20)	0.84 (0.70 to 1.01)	0.07
**Other oral menopausal treatment**						
Tibolone	177 / 781	0.99 (0.82 to 1.18)	119 / 463	1.05 (0.83 to 1.32)	1.01 (0.87 to 1.16)	0.9
Raloxifene	91 / 273	1.58 (1.22 to 2.06)	50 / 151	1.32 (0.92 to 1.91)	1.49 (1.20 to 1.84)	<0.001

CEE conjugated equine oestrogen; E2 oestradiol; MPA medroxyprogesterone acetate

*Odds ratios are based on cases and controls matched by age and practice and adjusted for smoking status, body mass index, family history of VTE, chronic and recent medical events, other medications and past exposures to hormones.

†other progestogens include norgestrel/levonorgestrel and drospirenone

eTable 9 ***Cases restricted to patients with a diagnosis of VTE on hospital or mortality records, or with associated anticoagulant prescriptions, and their controls:*** *Numbers of cases and controls and adjusted odds ratios by database and combined odds ratios*

	QResearch		CPRD		Combined analysis	
	N of cases/ controls	Adjusted Odds ratio (95%CI)	N of cases/ controls	Adjusted Odds ratio (95%CI)		
Total N	39630 \ 197310		9696 \ 44440			
**ANY HRT**	2684 \ 10375	1.47 (1.39 to 1.55)	791 \ 2565	1.61 (1.46 to 1.79)	1.50 (1.43 to 1.57)	<0.001
**ORAL PREPARATIONS**	2339 \ 8053	1.66 (1.58 to 1.76)	693 \ 2083	1.76 (1.58 to 1.97)	1.68 (1.60 to 1.77)	<0.001
**Oestrogen-only**						
CEE	552 \ 1908	1.53 (1.38 to 1.70)	163 \ 504	1.53 (1.25 to 1.88)	1.53 (1.39 to 1.68)	<0.001
E2	369 \ 1398	1.30 (1.14 to 1.47)	106 \ 331	1.49 (1.16 to 1.92)	1.33 (1.19 to 1.50)	<0.001
**Combined with progestogen**						
CEE MPA	385 \ 1031	2.43 (2.13 to 2.76)	102 \ 263	2.37 (1.83 to 3.08)	2.42 (2.15 to 2.71)	<0.001
CEE Norgestrel	268 \ 937	1.75 (1.51 to 2.03)	86 \ 247	2.00 (1.51 to 2.63)	1.80 (1.58 to 2.05)	<0.001
E2 MPA	35 \ 148	1.49 (1.00 to 2.20)	8 \ 39	1.26 (0.54 to 2.93)	1.44 (1.01 to 2.06)	0.04
E2 Dydrogesterone	69 \ 368	1.15 (0.87 to 1.51)	20 \ 90	1.23 (0.72 to 2.11)	1.16 (0.91 to 1.49)	0.2
E2 Norethisterone	646 \ 2167	1.82 (1.65 to 2.00)	201 \ 593	1.88 (1.56 to 2.27)	1.83 (1.68 to 2.00)	<0.001
E2 other progestogens†	15 \ 96	0.98 (0.56 to 1.74)	7 \ 16	2.34 (0.87 to 6.33)	1.22 (0.74 to 1.99)	0.4
**TRANSDERMAL PREPARATIONS**	394 \ 2507	0.82 (0.73 to 0.92)	110 \ 500	1.02 (0.81 to 1.29)	0.85 (0.77 to 0.95)	0.003
E2 only	304 \ 1887	0.83 (0.73 to 0.95)	85 \ 406	0.94 (0.72 to 1.23)	0.85 (0.76 to 0.96)	0.008
E2 combined	90 \ 620	0.78 (0.61 to 0.98)	25 \ 94	1.41 (0.85 to 2.32)	0.87 (0.70 to 1.07)	0.2
**OTHER ORAL MENOPAUSAL TREATMENT**						
Tibolone	149 \ 874	0.95 (0.79 to 1.15)	47 \ 235	0.93 (0.65 to 1.32)	0.95 (0.80 to 1.12)	0.5
Raloxifene	83 \ 335	1.32 (1.01 to 1.71)	31 \ 73	2.08 (1.28 to 3.38)	1.46 (1.16 to 1.84)	0.001

CEE conjugated equine oestrogen; E2 oestradiol; MPA medroxyprogesterone acetate

*Odds ratios are based on cases and controls matched by age and practice and adjusted for smoking status, body mass index, family history of VTE, chronic and recent medical events, other medications and past exposures to hormones.

†other progestogens include norgestrel/levonorgestrel and drospirenone

eTable 10 ***Cases and controls without chronic conditions or recent medical events:*** *Numbers of cases and controls and adjusted odds ratios by database and combined odds ratios*

	QResearch		CPRD		Combined analysis	
	N of cases/ controls	Adjusted Odds ratio (95%CI)	N of cases/ controls	Adjusted Odds ratio (95%CI)		
Total N	17932 / 56747		8839 / 25931			
**Any HRT**	1609 / 3757	1.62 (1.51 to 1.74)	810 / 1700	1.53 (1.38 to 1.70)	1.59 (1.50 to 1.69)	<0.001
**Oral preparations**	1382 / 2980	1.77 (1.64 to 1.91)	715 / 1351	1.76 (1.58 to 1.96)	1.77 (1.66 to 1.88)	<0.001
**Oestrogen-only**						
CEE	315 / 660	1.57 (1.35 to 1.82)	165 / 252	1.78 (1.42 to 2.22)	1.63 (1.44 to 1.85)	<0.001
E2	191 / 449	1.35 (1.12 to 1.63)	101 / 239	1.06 (0.82 to 1.37)	1.25 (1.07 to 1.45)	0.004
**Combined with progestogen**						
CEE MPA	233 / 393	2.55 (2.13 to 3.04)	110 / 180	2.26 (1.74 to 2.94)	2.45 (2.12 to 2.84)	<0.001
CEE Norgestrel	194 / 457	1.84 (1.53 to 2.21)	110 / 171	2.73 (2.09 to 3.55)	2.09 (1.80 to 2.43)	<0.001
E2 MPA	24 / 45	2.09 (1.22 to 3.58)	14 / 34	1.56 (0.79 to 3.09)	1.87 (1.23 to 2.85)	0.004
E2 Dydrogesterone	44 / 145	1.21 (0.85 to 1.74)	21 / 68	1.10 (0.65 to 1.86)	1.18 (0.87 to 1.58)	0.3
E2 Norethisterone	369 / 790	1.96 (1.71 to 2.25)	189 / 393	1.81 (1.49 to 2.20)	1.91 (1.71 to 2.14)	<0.001
E2 other progestogens†	12 / 41	1.13 (0.57 to 2.23)	5 / 14	1.37 (0.45 to 4.14)	1.19 (0.67 to 2.12)	0.6
**Transdermal preparations**	259 / 853	1.04 (0.89 to 1.20)	105 / 363	0.78 (0.61 to 0.99)	0.95 (0.84 to 1.08)	0.5
E2 only	195 / 612	1.05 (0.88 to 1.25)	84 / 276	0.79 (0.61 to 1.03)	0.96 (0.83 to 1.11)	0.6
E2 combined	64 / 241	0.99 (0.74 to 1.32)	21 / 87	0.74 (0.44 to 1.26)	0.92 (0.71 to 1.19)	0.5
**Other oral menopausal treatment**						
Tibolone	75 / 319	0.84 (0.65 to 1.10)	48 / 144	1.11 (0.78 to 1.59)	0.93 (0.75 to 1.15)	0.5
Raloxifene	28 / 70	1.36 (0.85 to 2.18)	18 / 40	1.20 (0.66 to 2.20)	1.30 (0.90 to 1.88)	0.2

CEE conjugated equine oestrogen; E2 oestradiol; MPA medroxyprogesterone acetate

*Odds ratios are based on cases and controls matched by age and practice and adjusted for smoking status, body mass index, family history of VTE, chronic and recent medical events, other medications and past exposures to hormones.

†other progestogens include norgestrel/levonorgestrel and drospirenone

eTable 11 ***Cases and controls aged 40 to 54:*** *Numbers of cases and controls and adjusted odds ratios by database and combined odds ratios*

	QResearch		CPRD		Combined analysis	
	N of cases/ controls	Adjusted Odds ratio (95%CI)	N of cases/ controls	Adjusted Odds ratio (95%CI)	Combined odds ratio (95% CI)	P-value
Total N	12128 / 60484		6467 / 29463			
**Any HRT**	1313 / 4759	1.42 (1.31 to 1.54)	697 / 2289	1.39 (1.24 to 1.55)	1.41 (1.32 to 1.50)	<0.001
**Oral preparations**	1125 / 3797	1.53 (1.41 to 1.66)	586 / 1842	1.51 (1.34 to 1.70)	1.52 (1.42 to 1.63)	<0.001
**Oestrogen-only**						
CEE	245 / 668	1.63 (1.37 to 1.93)	121 / 320	1.52 (1.18 to 1.95)	1.59 (1.38 to 1.83)	<0.001
E2	234 / 735	1.27 (1.07 to 1.50)	125 / 360	1.26 (0.99 to 1.60)	1.26 (1.10 to 1.45)	0.001
**Combined with progestogen**						
CEE MPA	103 / 310	2.15 (1.67 to 2.77)	29 / 119	1.18 (0.73 to 1.89)	1.88 (1.51 to 2.35)	<0.001
CEE Norgestrel	203 / 656	1.84 (1.54 to 2.21)	104 / 319	2.01 (1.57 to 2.59)	1.90 (1.64 to 2.20)	<0.001
E2 MPA	19 / 77	1.49 (0.86 to 2.59)	12 / 57	0.99 (0.49 to 1.98)	1.27 (0.82 to 1.96)	0.3
E2 Dydrogesterone	55 / 264	1.33 (0.97 to 1.85)	27 / 132	1.05 (0.65 to 1.69)	1.24 (0.95 to 1.62)	0.1
E2 Norethisterone	254 / 1030	1.39 (1.19 to 1.63)	163 / 509	1.69 (1.37 to 2.09)	1.49 (1.31 to 1.69)	<0.001
E2 other progestogens†	12 / 57	1.38 (0.70 to 2.72)	5 / 26	1.16 (0.42 to 3.19)	1.31 (0.74 to 2.30)	0.4
**Transdermal preparations**	236 / 1094	1.00 (0.85 to 1.17)	123 / 478	0.92 (0.72 to 1.16)	0.97 (0.85 to 1.11)	0.7
E2 only	184 / 769	1.05 (0.88 to 1.26)	98 / 361	0.92 (0.70 to 1.19)	1.01 (0.86 to 1.17)	0.9
E2 combined	52 / 325	0.88 (0.64 to 1.22)	25 / 117	0.94 (0.56 to 1.57)	0.90 (0.68 to 1.18)	0.4
**Other oral menopausal treatment**						
Tibolone	51 / 194	1.11 (0.78 to 1.59)	38 / 101	1.75 (1.13 to 2.71)	1.33 (1.01 to 1.75)	0.04
Raloxifene	7 / 15	3.11 (1.15 to 8.46)	<5 cases	N/A	3.11 (1.15 to 8.46)[Q]	0.05

CEE conjugated equine oestrogen; E2 oestradiol; MPA medroxyprogesterone acetate

*Odds ratios are based on cases and controls matched by age and practice and adjusted for smoking status, body mass index, family history of VTE, chronic and recent medical events, other medications and past exposures to hormones.

†other progestogens include norgestrel/levonorgestrel and drospirenone

[Q] Results based on QResearch analysis only

eTable 12 ***Cases and controls aged 55 to 64:*** *Numbers of cases and controls and adjusted odds ratios by database and combined odds ratios*

	QResearch		CPRD		Combined analysis	
	N of cases/ controls	Adjusted Odds ratio (95%CI)	N of cases/ controls	Adjusted Odds ratio (95%CI)	Combined odds ratio (95% CI)	P-value
Total N	11721 / 58392		6566 / 30652			
**ANY HRT**	1552 / 6125	1.49 (1.38 to 1.60)	874 / 3071	1.53 (1.38 to 1.69)	1.50 (1.42 to 1.59)	<0.001
**ORAL PREPARATIONS**	1335 / 4771	1.69 (1.57 to 1.82)	759 / 2473	1.68 (1.52 to 1.87)	1.69 (1.59 to 1.80)	<0.001
**Oestrogen-only**						
CEE	292 / 1083	1.35 (1.16 to 1.56)	179 / 541	1.49 (1.22 to 1.83)	1.40 (1.24 to 1.57)	<0.001
E2	181 / 720	1.24 (1.03 to 1.49)	95 / 366	1.17 (0.89 to 1.53)	1.22 (1.05 to 1.42)	0.01
**Combined with progestogen**						
CEE MPA	254 / 703	2.54 (2.16 to 2.99)	138 / 404	2.02 (1.61 to 2.53)	2.35 (2.06 to 2.68)	<0.001
CEE Norgestrel	124 / 546	1.50 (1.21 to 1.85)	85 / 249	2.06 (1.56 to 2.74)	1.68 (1.42 to 2.00)	<0.001
E2 MPA	21 / 88	1.77 (1.05 to 3.00)	9 / 38	1.47 (0.66 to 3.28)	1.68 (1.08 to 2.60)	0.02
E2 Dydrogesterone	31 / 192	1.07 (0.71 to 1.62)	19 / 97	1.26 (0.73 to 2.18)	1.14 (0.82 to 1.58)	0.4
E2 Norethisterone	420 / 1387	2.05 (1.81 to 2.33)	228 / 763	1.88 (1.58 to 2.24)	1.99 (1.80 to 2.21)	<0.001
E2 other progestogens†	12 / 52	1.46 (0.75 to 2.85)	6 / 15	2.40 (0.82 to 7.01)	1.68 (0.95 to 2.96)	0.07
**TRANSDERMAL PREPARATIONS**	235 / 1445	0.82 (0.70 to 0.96)	123 / 619	0.92 (0.73 to 1.15)	0.85 (0.75 to 0.97)	0.01
E2 only	183 / 1072	0.82 (0.69 to 0.98)	94 / 472	0.91 (0.71 to 1.18)	0.85 (0.74 to 0.98)	0.03
E2 combined	52 / 373	0.77 (0.56 to 1.06)	29 / 147	0.92 (0.58 to 1.45)	0.81 (0.63 to 1.06)	0.1
**OTHER ORAL MENOPAUSAL TREATMENT**						
Tibolone	98 / 509	1.16 (0.91 to 1.47)	65 / 290	1.02 (0.74 to 1.41)	1.11 (0.91 to 1.34)	0.3
Raloxifene	25 / 92	1.28 (0.78 to 2.12)	17 / 55	1.41 (0.74 to 2.68)	1.33 (0.89 to 1.97)	0.2

CEE conjugated equine oestrogen; E2 oestradiol; MPA medroxyprogesterone acetate

*Odds ratios are based on cases and controls matched by age and practice and adjusted for smoking status, body mass index, family history of VTE, chronic and recent medical events, other medications and past exposures to hormones.

†other progestogens include norgestrel/levonorgestrel and drospirenone

eTable 13 ***Cases and controls aged 65 to 79:*** *Numbers of cases and controls and adjusted odds ratios by database and combined odds ratios*

	QResearch		CPRD		Combined analysis	
	N of cases/ controls	Adjusted Odds ratio (95%CI)	N of cases/ controls	Adjusted Odds ratio (95%CI)	Combined odds ratio (95% CI)	P-value
Total N	28288 / 140666		15226 / 71837			
**ANY HRT**	904 / 3720	1.38 (1.27 to 1.50)	455 / 1706	1.38 (1.22 to 1.55)	1.38 (1.29 to 1.47)	<0.001
**ORAL PREPARATIONS**	747 / 2770	1.55 (1.42 to 1.70)	363 / 1285	1.49 (1.30 to 1.70)	1.53 (1.42 to 1.65)	<0.001
**Oestrogen-only**						
CEE	249 / 941	1.45 (1.24 to 1.68)	122 / 447	1.37 (1.10 to 1.71)	1.42 (1.26 to 1.61)	<0.001
E2	96 / 421	1.18 (0.93 to 1.50)	40 / 179	0.90 (0.61 to 1.32)	1.10 (0.90 to 1.35)	0.4
**Combined with progestogen**						
CEE MPA	144 / 425	2.02 (1.65 to 2.48)	72 / 209	2.15 (1.60 to 2.88)	2.06 (1.74 to 2.44)	<0.001
CEE Norgestrel	55 / 230	1.44 (1.05 to 1.96)	43 / 107	2.36 (1.61 to 3.46)	1.75 (1.37 to 2.22)	<0.001
E2 MPA	9 / 32	1.82 (0.84 to 3.96)	<5 cases	N/A	1.82 (0.84 to 3.96) [Q]	0.1
E2 Dydrogesterone	15 / 64	1.63 (0.90 to 2.94)	5 / 30	1.25 (0.46 to 3.40)	1.52 (0.92 to 2.53)	0.1
E2 Norethisterone	174 / 626	1.70 (1.42 to 2.04)	76 / 298	1.33 (1.00 to 1.78)	1.59 (1.36 to 1.85)	<0.001
E2 other progestogens†	5 / 31	1.20 (0.45 to 3.18)	<5 cases	N/A	1.20 (0.45 to 3.18) [Q]	0.2
**TRANSDERMAL PREPARATIONS**	169 / 980	0.92 (0.78 to 1.10)	96 / 428	1.07 (0.83 to 1.36)	0.97 (0.84 to 1.12)	0.7
E2 only	136 / 805	0.90 (0.74 to 1.09)	86 / 371	1.09 (0.84 to 1.41)	0.96 (0.82 to 1.12)	0.6
E2 combined	33 / 175	1.02 (0.69 to 1.52)	10 / 57	0.92 (0.44 to 1.89)	1.00 (0.70 to 1.41)	1
**OTHER ORAL MENOPAUSAL TREATMENT**						
Tibolone	75 / 515	0.77 (0.60 to 0.99)	41 / 250	0.82 (0.57 to 1.18)	0.79 (0.64 to 0.97)	0.02
Raloxifene	82 / 312	1.43 (1.10 to 1.86)	47 / 147	1.51 (1.04 to 2.20)	1.46 (1.18 to 1.81)	<0.001

CEE conjugated equine oestrogen; E2 oestradiol; MPA medroxyprogesterone acetate

*Odds ratios are based on cases and controls matched by age and practice and adjusted for smoking status, body mass index, family history of VTE, chronic and recent medical events, other medications and past exposures to hormones.

†other progestogens include norgestrel/levonorgestrel and drospirenone

[Q] Results based on QResearch analysis only

eTable 14 ***Cases and controls with body mass index < 25kg/m²:*** *Numbers of cases and controls and adjusted odds ratios by database and combined odds ratios*

	QResearch		CPRD		Combined analysis	
	N of cases/ controls	Adjusted Odds ratio (95%CI)	N of cases/ controls	Adjusted Odds ratio (95%CI)	Combined odds ratio (95% CI)	P-value
Total N	11160 / 22954		5955 / 11539			
**ANY HRT**	1011 / 1602	1.41 (1.27 to 1.56)	493 / 800	1.34 (1.15 to 1.55)	1.39 (1.27 to 1.51)	<0.001
**ORAL PREPARATIONS**	850 / 1246	1.51 (1.35 to 1.69)	421 / 648	1.48 (1.26 to 1.74)	1.50 (1.37 to 1.64)	<0.001
**Oestrogen-only**						
CEE	181 / 274	1.24 (0.99 to 1.55)	97 / 142	1.24 (0.91 to 1.70)	1.24 (1.04 to 1.49)	0.02
E2	110 / 172	1.17 (0.88 to 1.55)	49 / 93	0.94 (0.62 to 1.43)	1.09 (0.86 to 1.38)	0.5
**Combined with progestogen**						
CEE MPA	137 / 151	2.15 (1.64 to 2.81)	68 / 94	2.18 (1.50 to 3.17)	2.16 (1.74 to 2.68)	<0.001
CEE Norgestrel	122 / 167	1.76 (1.34 to 2.32)	60 / 88	1.98 (1.35 to 2.89)	1.83 (1.47 to 2.29)	<0.001
E2 MPA	15 / 23	1.65 (0.80 to 3.41)	5 / 13	0.67 (0.21 to 2.09)	1.27 (0.69 to 2.35)	0.4
E2 Dydrogesterone	34 / 82	1.09 (0.70 to 1.69)	20 / 29	1.61 (0.82 to 3.19)	1.22 (0.84 to 1.76)	0.3
E2 Norethisterone	240 / 358	1.65 (1.37 to 2.00)	120 / 182	1.57 (1.18 to 2.09)	1.63 (1.39 to 1.91)	<0.001
E2 other progestogens†	11 / 19	1.34 (0.59 to 3.00)	<5 cases	N/A	1.34 (0.59 to 3.00) [Q]	0.7
**TRANSDERMAL PREPARATIONS**	180 / 380	1.05 (0.85 to 1.30)	81 / 164	0.86 (0.63 to 1.19)	0.99 (0.83 to 1.18)	0.9
E2 only	133 / 269	1.06 (0.83 to 1.36)	62 / 108	1.02 (0.70 to 1.48)	1.05 (0.85 to 1.29)	0.7
E2 combined	47 / 111	1.02 (0.69 to 1.50)	19 / 56	0.56 (0.30 to 1.04)	0.86 (0.62 to 1.20)	0.4
**OTHER ORAL MENOPAUSAL TREATMENT**						
Tibolone	61 / 115	1.23 (0.86 to 1.76)	34 / 63	1.07 (0.65 to 1.76)	1.17 (0.87 to 1.57)	0.3
Raloxifene	41 / 60	1.19 (0.74 to 1.90)	22 / 20	2.00 (0.97 to 4.13)	1.39 (0.94 to 2.06)	0.1

CEE conjugated equine oestrogen; E2 oestradiol; MPA medroxyprogesterone acetate

*Odds ratios are based on cases and controls matched by age and practice and adjusted for smoking status, body mass index, family history of VTE, chronic and recent medical events, other medications and past exposures to hormones.

†other progestogens include norgestrel/levonorgestrel and drospirenone

[Q] Results based on QResearch analysis only

eTable 15 ***Cases and controls with body mass index 25 to 30kg/m²:*** *Numbers of cases and controls and adjusted odds ratios by database and combined odds ratios*

	QResearch		CPRD		Combined analysis	
	N of cases/ controls	Adjusted Odds ratio (95%CI)	N of cases/ controls	Adjusted Odds ratio (95%CI)	Combined odds ratio (95% CI)	P-value
Total N	11600 / 21056		6923 / 12529			
**ANY HRT**	906 / 1127	1.58 (1.42 to 1.76)	534 / 668	1.59 (1.37 to 1.84)	1.58 (1.45 to 1.73)	<0.001
**ORAL PREPARATIONS**	772 / 850	1.82 (1.61 to 2.05)	453 / 526	1.75 (1.49 to 2.06)	1.79 (1.63 to 1.97)	<0.001
**Oestrogen-only**						
CEE	202 / 192	2.03 (1.61 to 2.55)	118 / 123	1.89 (1.40 to 2.56)	1.98 (1.65 to 2.37)	<0.001
E2	121 / 151	1.41 (1.07 to 1.86)	68 / 87	1.52 (1.04 to 2.21)	1.45 (1.16 to 1.81)	0.001
**Combined with progestogen**						
CEE MPA	123 / 131	2.40 (1.80 to 3.18)	70 / 64	2.63 (1.75 to 3.97)	2.47 (1.96 to 3.12)	<0.001
CEE Norgestrel	76 / 92	1.62 (1.15 to 2.29)	57 / 67	1.90 (1.25 to 2.88)	1.73 (1.33 to 2.26)	<0.001
E2 MPA	16 / 23	1.49 (0.72 to 3.10)	7 / 7	3.27 (1.03 to 10.40)	1.87 (1.01 to 3.46)	0.05
E2 Dydrogesterone	25 / 34	1.71 (0.95 to 3.06)	11 / 24	0.82 (0.34 to 1.95)	1.36 (0.84 to 2.21)	0.2
E2 Norethisterone	203 / 214	1.80 (1.45 to 2.24)	120 / 152	1.48 (1.10 to 1.98)	1.68 (1.41 to 2.00)	<0.001
E2 other progestogens†	6 / 13	1.01 (0.34 to 2.99)	<5 cases	N/A	1.01 (0.34 to 2.99) [Q]	0.8
**TRANSDERMAL PREPARATIONS**	154 / 296	0.89 (0.71 to 1.11)	83 / 146	0.99 (0.72 to 1.37)	0.92 (0.77 to 1.11)	0.4
E2 only	123 / 235	0.87 (0.67 to 1.11)	68 / 131	0.87 (0.61 to 1.24)	0.87 (0.71 to 1.06)	0.2
E2 combined	31 / 61	0.97 (0.59 to 1.60)	15 / 15	2.41 (1.03 to 5.64)	1.23 (0.80 to 1.89)	0.4
**OTHER ORAL MENOPAUSAL TREATMENT**						
Tibolone	41 / 116	0.62 (0.42 to 0.93)	38 / 62	1.25 (0.77 to 2.05)	0.82 (0.60 to 1.12)	0.2
Raloxifene	21 / 36	0.94 (0.51 to 1.73)	16 / 15	1.86 (0.81 to 4.26)	1.19 (0.73 to 1.95)	0.5

CEE conjugated equine oestrogen; E2 oestradiol; MPA medroxyprogesterone acetate

*Odds ratios are based on cases and controls matched by age and practice and adjusted for smoking status, body mass index, family history of VTE, chronic and recent medical events, other medications and past exposures to hormones.

†other progestogens include norgestrel/levonorgestrel and drospirenone

[Q] Results based on QResearch analysis only

eTable 16 ***Cases and controls with body mass index > 30kg/m²:*** *Numbers of cases and controls and adjusted odds ratios by database and combined odds ratios*

	QResearch		CPRD		Combined analysis	
	N of cases/ controls	Adjusted Odds ratio (95%CI)	N of cases/ controls	Adjusted Odds ratio (95%CI)	Combined odds ratio (95% CI)	P-value
Total N	13254 / 22034		7053 / 11305			
**ANY HRT**	686 / 837	1.46 (1.29 to 1.65)	402 / 444	1.59 (1.34 to 1.89)	1.50 (1.36 to 1.66)	<0.001
**ORAL PREPARATIONS**	590 / 655	1.60 (1.40 to 1.83)	333 / 344	1.75 (1.46 to 2.11)	1.65 (1.48 to 1.84)	<0.001
**Oestrogen-only**						
CEE	148 / 164	1.50 (1.16 to 1.94)	84 / 92	1.33 (0.93 to 1.90)	1.44 (1.17 to 1.78)	<0.001
E2	124 / 156	1.29 (0.99 to 1.69)	62 / 74	1.40 (0.95 to 2.07)	1.32 (1.06 to 1.65)	0.01
**Combined with progestogen**						
CEE MPA	82 / 72	2.22 (1.55 to 3.17)	43 / 32	3.59 (2.12 to 6.06)	2.58 (1.92 to 3.46)	<0.001
CEE Norgestrel	50 / 67	1.42 (0.95 to 2.13)	36 / 36	1.75 (1.04 to 2.94)	1.54 (1.12 to 2.11)	0.008
E2 MPA	6 / 11	0.91 (0.31 to 2.65)	5 / 6	1.66 (0.45 to 6.09)	1.16 (0.51 to 2.64)	0.7
E2 Dydrogesterone	16 / 33	0.90 (0.46 to 1.74)	9 / 13	1.24 (0.48 to 3.19)	1.00 (0.58 to 1.72)	1
E2 Norethisterone	159 / 150	2.07 (1.60 to 2.68)	89 / 89	2.05 (1.45 to 2.90)	2.06 (1.68 to 2.54)	<0.001
E2 other progestogens†	<5 cases	N/A	<5 cases	N/A	N/A	
**TRANSDERMAL PREPARATIONS**	109 / 195	0.95 (0.73 to 1.24)	75 / 106	0.98 (0.69 to 1.40)	0.96 (0.78 to 1.19)	0.7
E2 only	89 / 152	1.02 (0.76 to 1.37)	59 / 82	1.04 (0.69 to 1.56)	1.02 (0.81 to 1.30)	0.8
E2 combined	20 / 43	0.70 (0.38 to 1.28)	16 / 24	0.79 (0.37 to 1.70)	0.73 (0.45 to 1.18)	0.2
**OTHER ORAL MENOPAUSAL TREATMENT**						
Tibolone	56 / 83	1.03 (0.69 to 1.52)	31 / 31	1.79 (0.99 to 3.22)	1.22 (0.88 to 1.69)	0.2
Raloxifene	12 / 15	1.32 (0.56 to 3.12)	10 / 11	1.43 (0.51 to 4.05)	1.37 (0.70 to 2.65)	0.4

CEE conjugated equine oestrogen; E2 oestradiol; MPA medroxyprogesterone acetate

*Odds ratios are based on cases and controls matched by age and practice and adjusted for smoking status, body mass index, family history of VTE, chronic and recent medical events, other medications and past exposures to hormones.

†other progestogens include norgestrel/levonorgestrel and drospirenone
